# Lateral root enriched *Massilia* associated with plant flowering in maize

**DOI:** 10.1186/s40168-024-01839-4

**Published:** 2024-07-09

**Authors:** Danning Wang, Xiaoming He, Marcel Baer, Klea Lami, Baogang Yu, Alberto Tassinari, Silvio Salvi, Gabriel Schaaf, Frank Hochholdinger, Peng Yu

**Affiliations:** 1 Emmy Noether Group Root Functional Biology, Institute of Crop Science and Resource Conservation (INRES), University of Bonn, Bonn, 53113 Germany; 2 Crop Functional Genomics, Institute of Crop Science and Resource Conservation (INRES), University of Bonn, Bonn, 53113 Germany; 3 Plant Nutrition, Institute of Crop Science and Resource Conservation (INRES), University of Bonn, Bonn, 53113 Germany; 4 Department of Agricultural and Food Sciences, University of Bologna, Bologna, 40127 Italy

**Keywords:** Lateral roots, Maize, *Massilia*, Rhizosphere microbiome, Root transcriptome

## Abstract

**Background:**

Beneficial associations between plants and soil microorganisms are critical for crop fitness and resilience. However, it remains obscure how microorganisms are assembled across different root compartments and to what extent such recruited microbiomes determine crop performance. Here, we surveyed the root transcriptome and the root and rhizosphere microbiome via RNA sequencing and full-length (V1–V9) 16S rRNA gene sequencing from genetically distinct monogenic root mutants of maize (*Zea mays* L.) under different nutrient-limiting conditions.

**Results:**

Overall transcriptome and microbiome display a clear assembly pattern across the compartments, i.e., from the soil through the rhizosphere to the root tissues. Co-variation analysis identified that genotype dominated the effect on the microbial community and gene expression over the nutrient stress conditions. Integrated transcriptomic and microbial analyses demonstrated that mutations affecting lateral root development had the largest effect on host gene expression and microbiome assembly, as compared to mutations affecting other root types. Cooccurrence and trans-kingdom network association analysis demonstrated that the keystone bacterial taxon *Massilia* (*Oxalobacteraceae*) is associated with root functional genes involved in flowering time and overall plant biomass. We further observed that the developmental stage drives the differentiation of the rhizosphere microbial assembly, especially the associations of the keystone bacteria *Massilia* with functional genes in reproduction. Taking advantage of microbial inoculation experiments using a maize early flowering mutant, we confirmed that *Massilia*-driven maize growth promotion indeed depends on flowering time.

**Conclusion:**

We conclude that specific microbiota supporting lateral root formation could enhance crop performance by mediating functional gene expression underlying plant flowering time in maize.

Video Abstract

**Supplementary Information:**

The online version contains supplementary material available at 10.1186/s40168-024-01839-4.

## Introduction

Plant roots interact with their environment via the rhizosphere, which is the narrow soil volume that is directly influenced by the properties and activities of host plants [1]. A single root comprises different tissues, including the epidermis, cortex, and stele [2] and hosts a wide variety of endophytic microorganisms [3]. These endophytes can be bacteria or fungi substantially supporting their host plants in the acquisition of soil water and nutrients [4]. The community of microorganisms and their genomic information constitute the microbiome that is found living in the root system and rhizosphere. The self-organization of the microbiome community associated with the root system and rhizosphere is shaped by a cascade of feedback loops between root development, microbial assembly, and soil properties [5]. Studies in *Arabidopsis*, rice, and maize have shown that the taxonomic composition of the root-inhabiting microbiome is strongly influenced by geography and soil types [6, 7]. Moreover, the plant genotype shapes the composition of the endophytic microbiome [8] likely by root exudates, which may act as signals for microbial recognition [9]. As a consequence of complex interactions between root, microbiome, and soil, the rhizosphere can be considered a self-organizing system whose emerging patterns cannot be understood by studying the individual components in isolation but only by a systemic approach in consideration of spatial resolution.

The complex 3D structured root system of cereals is essential for the efficient uptake of water and minerals and thus for their productivity [10]. Therefore, root systems offer great potential for crop improvement in unfavorable environments [11]. The root system architecture is shaped by intrinsic genetically encoded regulators and enormous developmental plasticity that allows continuous adjustment of the root stock to fluctuating environmental conditions [12–14]. Mutant analyses have revealed that root-type-specific genetic regulators determine root system architecture in cereals [15, 16]. A plethora of studies have highlighted lateral roots and root hairs as the major determinants of root system architecture [17]. They substantially increase the root surface and are therefore instrumental for foraging nutrients and water resources in crops [18, 19] and have great potential in adaptation to unfavorable conditions such as nutrient-deficient soil. Lateral roots are initiated post-embryonically from pericycle cells deep inside all root types, while root hairs are tubular extensions of epidermal cells at the root surface [19, 20]. Molecular cloning of genes underlying maize root formation has demonstrated that key elements of auxin signal transduction, such as LOB domain and Aux/IAA proteins, are instrumental for seminal, shoot-borne, and lateral root initiation [16]. Moreover, genetic analyses have demonstrated that genes related to exocytotic vesicle docking, cell wall loosening, and cellulose synthesis and organization control root hair elongation and/or initiation [16].

Genotypes with sparsely and long-distributed lateral roots are optimal for nitrate acquisition, whereas genotypes with densely spaced and short lateral roots are optimal for phosphorus acquisition in maize [21]. Recent results in maize have indicated that genotypes with higher lateral root branching density display significantly increased phosphorus acquisition under phosphorus-deficient conditions [22]. In contrast, maize genotypes with few and long lateral roots are more competent for nitrogen uptake than genotypes with many and short lateral roots under suboptimal nitrogen concentrations in soil [23]. Thus, the availability of soil nutrients determines compensatory growth and patterning of lateral roots along the parental root axes. The participation of root hairs in the uptake of nutrients has been widely acknowledged in crop species such as maize, wheat, and barley [18]. Root hair formation has been suggested to have a major impact on plant performance especially under unfavourable conditions such as drought or nutrient deficiency. Thus, overall root system architecture shaped by lateral roots and root hairs is an adaptation to unfavorable conditions such as nutrient-deficient soil.

Emerging lateral roots and root hairs are important sites for the release of exudates to the rhizosphere. A broad range of substrates and signaling molecules are secreted by roots to communicate with rhizosphere-inhabiting microorganisms [24–27]. The release of easily decomposable exudates by roots leads to higher microbial density and activity in the rhizosphere compared to the bulk soil [1]. Thus, root hairs are also a major determinant of both rhizosphere formation, i.e., the proportion of soil modified by the roots [28], and functioning, i.e., the metabolic reactions taking place at the root-soil interface [29]. Several publications indicate that plant growth-promoting rhizobacteria are able to manipulate primary root development [30, 31] but also lateral root formation [32–34] in *Arabidopsis thaliana*. In crops, root- and rhizosphere-associated microbiota contribute to alleviating overall plant nutrient stress [35, 36]. Nevertheless, the cross-kingdom interplay between plants and microbes at the root-soil interface for structuring rhizosphere-associated microbial communities and their potential impact on plant growth and nutrient acquisition has so far received little attention in crops.

In this study, we elucidated whether and how consistent patterns between root development and associated microbiome have emerged by employing genetically distinct monogenic root mutants in a model crop maize. We profiled root transcriptome and root-/rhizosphere-associated microbiome community assemblage using RNA sequencing and full-length (V1–V9) 16S rRNA gene sequencing. We assessed trans-kingdom network associations and how specific bacteria are assembled spatially through different root types by interacting with plant genes. Understanding how root traits modulate their microbiome, and the influence of plant-microbe association on plant development provides novel insights into the establishment of beneficial host–microbiome associations in enhancing tolerance to environmental constraints.

## Results

### The root transcriptome associates with bacterial community assemblage across root compartments

**Fig. 1:**
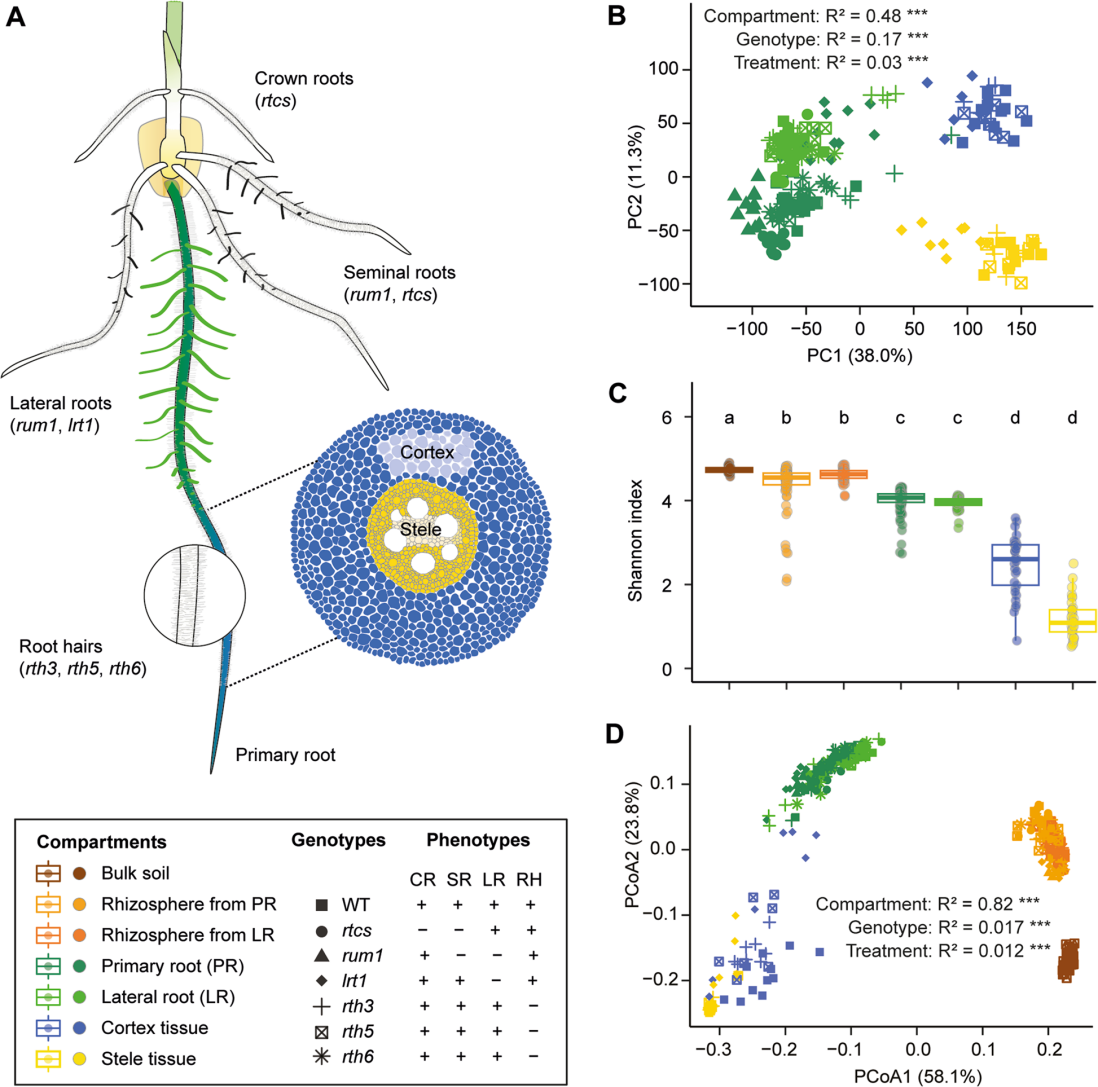
Overall gene expression pattern and bacterial diversity across spatial compartments. **A** Schematic illustration of maize seedling root system consisting of different root types and tissue (stele and cortex) patterning along the primary root. The cloned genes with known functions affecting root phenotypes are highlighted in brackets. The meristematic and elongation zones were removed during the sampling. The differentiation zone with root hairs and lateral root primordia was physically peeled off to separate the stele and cortex tissue. The differentiation zone with emerged lateral roots was dissected and separated as the lateral roots and primary root without lateral roots. *rum1*, *rootless with undetectable meristem 1*; *rtcs*, *rootless concerning crown and seminal roots*; *lrt1*, *lateral rootless 1*; *rth*, *roothairless*. **B** Principal component analysis (PCA) illustrating the transcriptomic shift across different root compartments along the primary root. **C** Spatial shift of bacterial α-diversity (Shannon’s diversity index) across the rhizosphere and root compartments along the primary root. Significances were indicated among different compartments by different letters (Benjamini-Hochberg adjusted *P* < 0.05, Kruskal-Wallis test, Dunn’s *post*-*hoc* test). Boxes span from the first to the third quartiles, center lines represent median values and whiskers show data lying within the 1.5 × interquartile range of lower and upper quartiles. Data points at the ends of whiskers represent outliers. **D** Principal coordinate analysis (PCoA) showing the dissimilarity of bacterial β-diversity across the rhizosphere and root compartments. For both bacterial and transcriptomic dissimilarity matrix data, the explained variance by compartments, genotype, and nutrient treatment conditions was assessed by permutational analysis of variance (PERMANOVA, *P* < 0.001). We did not label different nutrient symbols due to the relatively small effect in comparison to the compartment and genotype. The sample sizes are indicated below: bulk soil (*n* = 42); rhizosphere from primary root (PR) (*n* = 78); rhizosphere from lateral root (LR) (*n* = 57); primary root (*n* = 84); lateral root (*n* = 60); cortex tissue (*n* = 33); stele tissue (*n* = 34)

To understand whether and how root development affects microbiome assembly across different compartments along a single root, we examined different root and rhizosphere compartments in the primary root (Fig. 1A). The root compartments included primary roots without lateral roots, lateral roots, as well as separated cortex and stele tissues of the root differentiation zone (Fig. 1A). Moreover, we also extracted the closely attached rhizospheres from both primary and lateral roots separately, and the bulk soil from the unplanted pot as the control. These compartments were taken from genetically diverse monogenic maize root mutants (*rum1*, *lrt1*, *rtcs*, *rth3*, *rth5*, and *rth6*) and their respective wild-type B73. We conducted the study in four biological replicates and under three nutrient conditions of natural soil: control soil with sufficient nutrients, low nitrogen soil, and low phosphorus soil. We performed transcriptome analysis via RNA sequencing for root compartments and conducted bacterial microbiome analysis via 16S full-length (V1–V9) rRNA gene sequencing for both the root and rhizosphere compartments (Fig. 1A).

We examined the overall gene expression pattern using 30,835 highly expressed genes (> 5 reads in > 4 samples) in at least one root tissue/nutrient treatment variant. Among all expressed genes, most (99%) of them displayed overlapping expression (Figure S1A), suggesting that genes are conserved and expressed across the root tissues. A principal component analysis (PCA) showed the transcript dissimilarities between different samples of root tissues and treatments. It illustrates the highest transcriptomic dissimilarity among different tissues (PERMANOVA, *R*^2^ = 0.48, *P* = 0.0001, Fig. 1B). Notably, the genotype was responsible for a much stronger variance (PERMANOVA, *R*^2^ = 0.17, *P* = 0.0001) of gene expression than nutrient treatment effect (PERMANOVA, *R*^2^ = 0.03, *P* = 0.0001). Moreover, primary root and lateral root transcriptomes differed substantially from the cortex and stele tissue-specific transcriptomes (Fig. 1B). Thus, these results indicated that root spatial compartmentation dominated the gene expression pattern of tissue specification over host genetics and abiotic stresses.

In parallel, we determined the microbiome diversity and spatial assembly from the soil through the rhizosphere to the root tissues. Prior to analysis, we filtered in total of 880 high-quality and abundant OTUs from 388 samples (Table S1). Among them, only 223 OTUs (25%) are conserved for all different compartments (Figure S1B). Bacterial microbiome richness (measured by α-diversity Shannon’s index) varied significantly among different compartments (Benjamini-Hochberg adjusted *P* < 0.05, Kruskal-Wallis test, Dunn’s *post*-*hoc* test) and reduced from bulk soil, through the rhizosphere and roots and further to different tissues (Fig. 1C). However, neither genotype (Figure S2A) nor nutrient treatment conditions (Figure S2B) had consistent effects on the α-diversity within compartments, except for the *rtcs* mutant with respect to the rhizosphere extracted from lateral roots (Figure S2A) and except for the rhizosphere obtained from lateral roots and primary roots of all possible genotypes under low phosphorus (Figure S2B). Such divergence might be explained by the substantial differences in the bacterial community of bulk soil under different nutrient conditions (Figure S2B). To investigate the impact of compartment, genotype, and nutrient condition on bacterial community composition, we performed a principal coordinate analysis (PCoA) for bacterial abundance. Overall, we observed a strong shift in bacterial community composition among different spatial compartments (PERMANOVA, *R*^2^ = 0.82, *P* = 0.0001) along a single root (Fig. 1D). Genotype (PERMANOVA, *R*^2^ = 0.02, *P* = 0.0001) and nutrient treatment conditions (PERMANOVA, *R*^2^ = 0.01, *P* = 0.0001) explained a small part but still significant variance of bacterial community composition. Taken together, these data suggest that root transcriptomic changes and spatial patterns of microbiome assembly synchronize with each other during root development.

### Lateral roots dramatically influence host gene expression and bacterial community composition

To explore the potential effect of root mutation and nutrient treatment conditions on host gene expression and microbial assembly, we first examined, within each compartment, the impact of genotype and nutrient treatment conditions on host gene expression and bacterial abundance. Using a PERMANOVA test, we observed that the genotype had consistently more impact (*R*^2^ = 0.29–0.52) on gene expression within each compartment than nutrient treatment conditions (*R*^2^ = 0.08–0.12) (Figures S3 and S5B, Table S2). In contrast, both genotype and nutrient treatment conditions had comparable impacts on bacterial composition (Figures S4 and S5A, Table S2). More specifically, the genotype explained more of the variance of the bacterial composition of the primary root rhizosphere as well as of the primary root, cortex, and stele (*R*^2^ = 0.16–0.27) than the soil nutrient status (*R*^2^ = 0.08–0.16). In contrast, for the lateral root and rhizosphere, the nutrient treatment condition (*R*^2^ = 0.15–0.27) explained more variance of the bacterial composition than the genotype (*R*^2^ = 0.11–0.14).

**Fig. 2:**
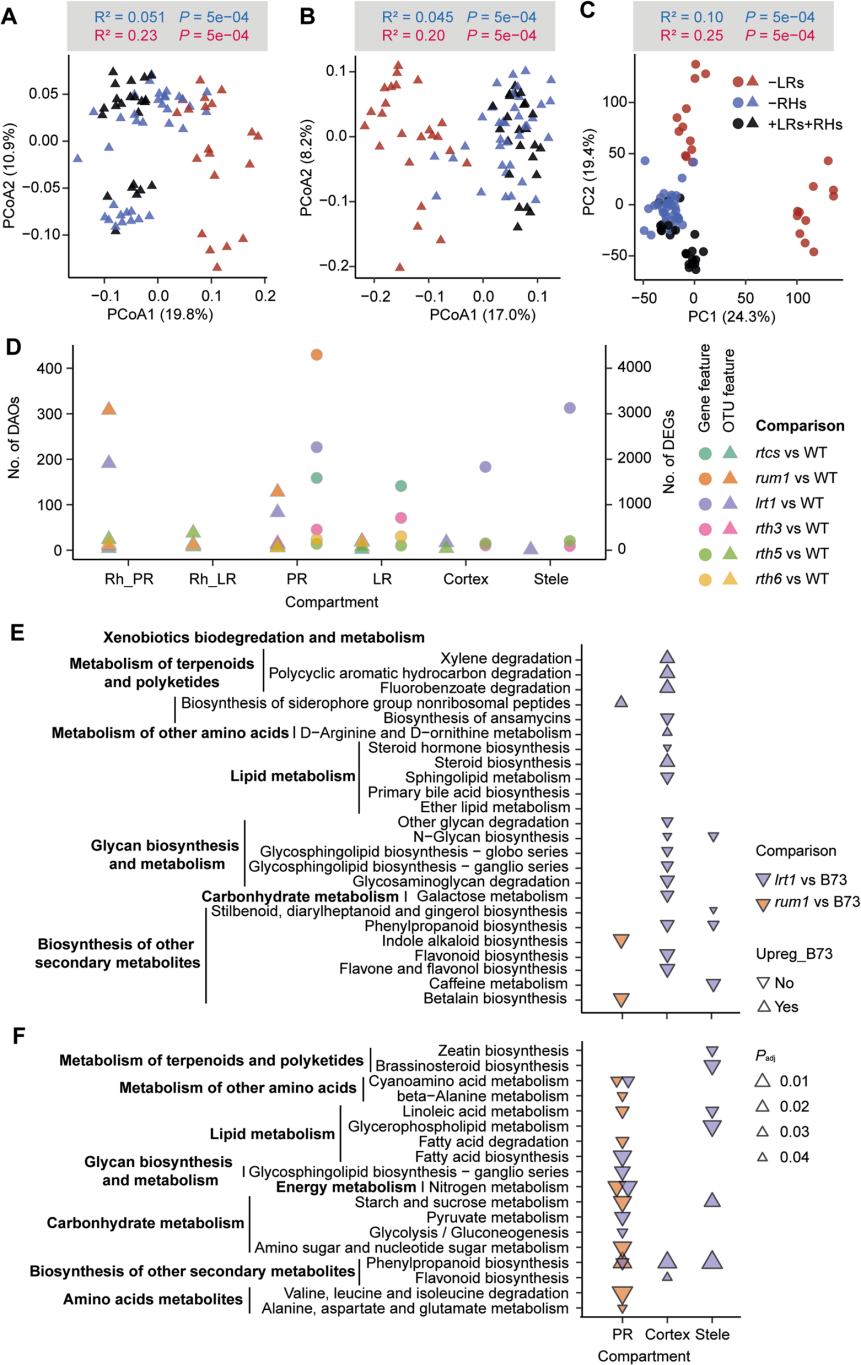
Lateral roots determine microbiome assemblage and transcriptomic changes across different compartments. Principal coordinate analysis (PCoA) showing the effects of root hairs and lateral root defects on the dissimilarity of bacterial β-diversity across 3 groups (WT: *n* = 23; Lateral root mutants: *n* = 20; Root hair mutants: *n* = 35) in the rhizosphere (**A**) and 3 groups (WT: *n* = 24; Lateral root mutants: *n* = 24; Root hair mutants: *n* = 36) in the root (**B**) compartments from primary root. **C** Principal component analysis (PCA) illustrating the effects of root hairs and lateral root defects on transcriptomic shift across 3 groups (WT: *n* = 24; lateral root mutants: *n* = 23; root hair mutants: *n* = 33) along the primary root. For both, the bacterial and transcriptomic dissimilarity matrix data, the explained variance by the effects from root hair and lateral roots were assessed by pair-wise permutational analysis of variance (PERMANOVA, *P* < 0.001). **D** Pair-wise comparison of differentially abundant OTUs (DAOs) and differentially expressed genes (DEGs) between each monogenic mutant and wild type (WT). The triangles and dots indicated the microbiome and transcriptome features respectively. Rh_PR, rhizosphere from the primary root; Rh_LR, rhizosphere from lateral root; PR, primary root; LR, lateral root. OTU, operational taxonomic unit. **E** Prediction of the functional potential of the bacterial microbiota using the PICRUSt tool from the lateral root mutants in comparison to the wild-type plants. Directions of triangles indicate up- and downregulation in mutant and wild-type B73. **F** Prediction of root metabolism using the KEGG pathway analysis from the lateral root mutants in comparison to the wild-type plant. The size of the triangles indicates the significance degree. The purple and orange color indicates the *lrt1* and *rum1* compared with wild-type B73 respectively

To specifically compare the mutation effects of root hairs and lateral roots on transcriptomic changes and microbiome assemblage within each compartment, we classified all genotypes into three groups: group 1 (WT and *rtcs*) with lateral roots and root hairs, group 2 (*rum1* and *lrt1*) with root hairs but no lateral roots and group 3 (*rth3*, *rth5*, and *rth6*) with lateral roots but no root hairs. As shown by PCoA and PCA, mutations that lead to lateral root defects (PERMANOVA, *R*^2^ = 0.20–0.25, *P* = 5e–04) have much stronger effects on the microbiome community composition and transcriptomic changes than mutations that result in impaired root hairs (PERMANOVA, *R*^2^ = 0.045–0.10, *P* = 5e–04) (Fig. 2A–C). Interestingly, we identified that there is also a significant (PERMANOVA, *R*^2^ = 0.21–0.31, *P* = 5e–04) genotype, i.e., *rum1* and *lrt1* effect within the lateral root mutation. We further performed pair-wise differential abundance/expression analysis for both bacteria and host genes between each mutant and wild-type B73. For the microbiome of primary roots and its rhizosphere, the genotypes *rum1* and *lrt1*, both defective in lateral root formation, displayed 100–300 more differentially abundant OTUs (operational taxonomic units) in comparison to the wild type. In contrast, genotypes defective in root hair formation (*rth3*, *rth5*, and *rth6*) showed much fewer changes (10–20 OTUs) (Fig. 2D). With respect to gene expression in the primary root, the lateral root mutants showed 2000–4300 differentially expressed genes in comparison to the wild type, while genotypes defective in root hair formation displayed < 500 differentially expressed genes (Fig. 2D). In particular, we identified > 1800 and > 3000 differentially expressed genes in the cortex and stele tissue of the *lrt1* mutant in comparison to the wild type (Fig. 2D). Overall, we found that lateral root mutation could confer to much stronger effect on host gene expression and microbial assembly than root hair mutation.

### Functional prediction of lateral root mutation-driven root microbiome and gene expressions

The PICRUSt approach was applied to evaluate the functional potential of microbial communities with a particular focus on the effect of lateral root mutation. We specifically focused on the differently abundant OTUs of two lateral root mutants, i.e., *rum1* and *lrt1* in comparison to the wild-type plants. Interestingly, a substantial proportion of metabolic pathways, e.g., biosynthesis of ansamycins, sphingolipid metabolism, glycan and carbohydrate metabolism, and biosynthesis of other secondary metabolites were exclusively enriched in cortex tissue of the *lrt1* mutant, but not in *rum1* or the other tissues (Fig. 2E, Table S3). Among the secondary metabolites, flavone and flavonol biosynthesis were the most differentially upregulated metabolic pathways in the *lrt1* cortex tissue (Fig. 2E). In contrast, biosynthesis of indole alkaloid and betalain was significantly enriched in the primary root of *rum1* mutant (Fig. 2E), which is consistent with the function of RUM1 as an Aux/IAA protein involved in auxin signaling. In the stele tissue, phenylpropanoid biosynthesis and caffeine metabolism are the only enriched pathways in the *lrt1* mutant (Fig. 2E). These predicted results demonstrated that specific LRT1 gene-encoded metabolic changes may confer beneficial root-microbe associations in the cortex of maize.

We next evaluated the tissue-specific metabolic potential of the differentially expressed genes between lateral root mutants and wild-type plants according to the KEGG pathways. In particular, we identified that nitrogen and cyanoamino acid metabolism pathways are significantly enriched in the primary root of both lateral rootless mutants, but neither in cortex nor stele tissue (Fig. 2F, Table S4). Specifically, the stele tissue of the *lrt1* mutant held great potential for amino acids and lipids metabolism in comparison to the *rum1* mutant (Fig. 2F, Table S4). Together, these functional prediction analyses highlight the enrichment of maize gene transcripts associated with nitrogen metabolism and potential roles of microbial flavonoids driven root–microbe associations along spatial root compartments.

### Lateral roots and their rhizosphere recruit highly complex bacterial networks

To further understand the bacteria-bacteria associations across different root and rhizosphere compartments, we next applied the co-occurrence network approach between bacterial OTUs within each compartment. To reduce the impact of rare OTUs, only OTUs with a relative abundance of > 0.1% in ≥ 10% of samples were kept for network construction using the SparCC algorithm for each compartment. Network correlation was calculated using the default centered log-ratio (CLR) transformed filtered bacterial table based on 100 bootstraps. Among these co-occurrence networks, the complexity of networks, total number of associations, and associated OTUs decreased from soil, via the rhizosphere, to the root, and to the tissues (Figures S6 and S7). We then calculated the hub score for each OTU in each network and nodes which were ranked in the top 10 were considered as keystone OTUs. We found that keystone OTUs belonging to phyla *Gemmatimodadetes*, *Planctomycetes*, and *Bacteroidetes* in the soil, keystone OTUs belonging to phylum *Proteobacteria* in the rhizosphere, and primary root and keystone OTUs belonging to phyla *Proteobacteria* and *Bacteroidetes* in the lateral root and cortex tissue (Table S5). Interestingly, we detected the two network hubs OTU3535 (*Massilia*) and OTU5737 (*Pseudoduganella*) belonging to *Oxalobacteraceae* in both lateral root and cortex tissue (Figure S6E, F).

**Fig. 3:**
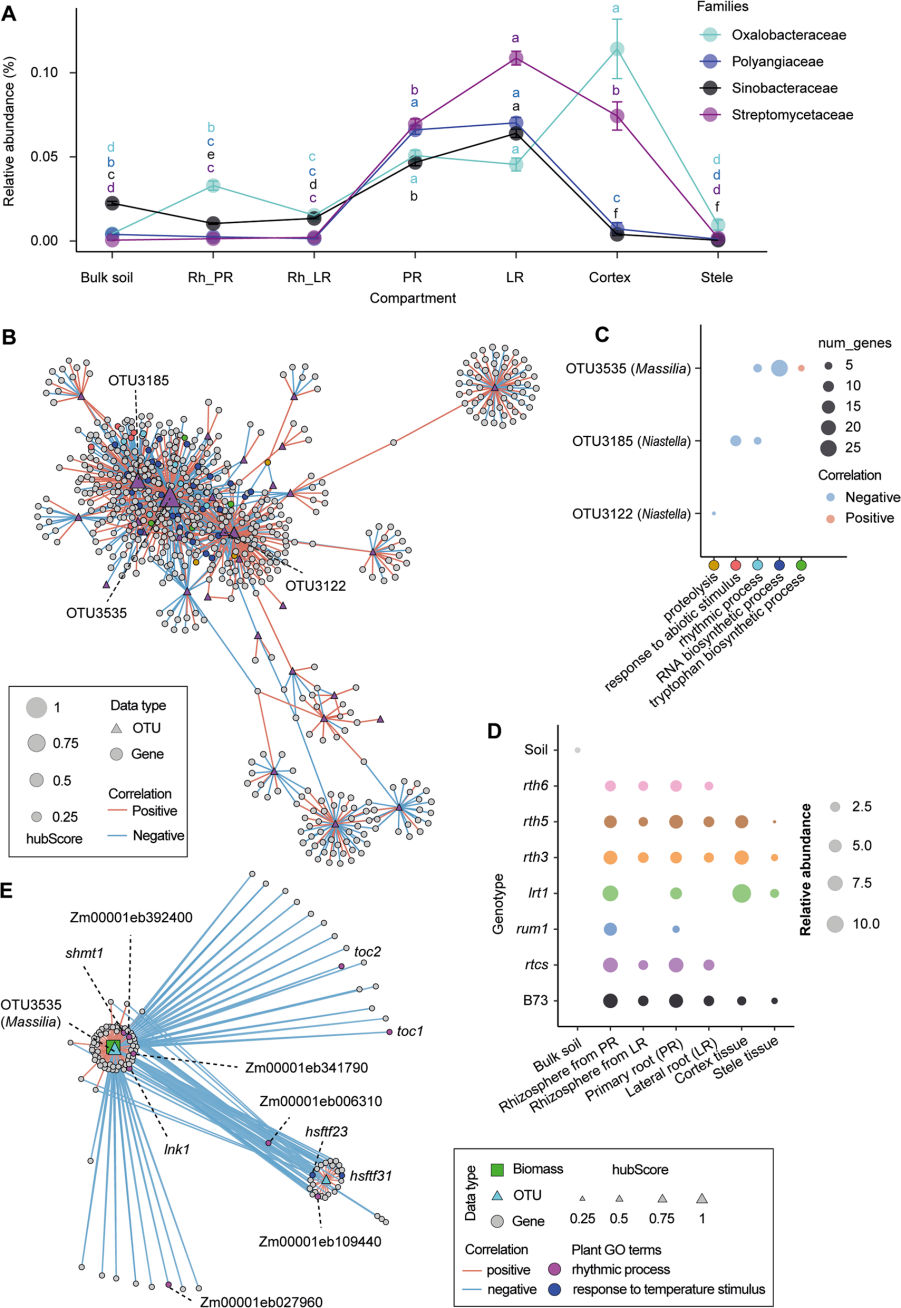
Trans-kingdom associations between root genes and bacterial OTUs in root microbiota. **A** Selective enrichment of different bacterial families from the bulk soil to the rhizosphere, root, and root tissues. Significances were indicated among different compartments by different letters for each family (Benjamini-Hochberg adjusted *P* < 0.05, Kruskal-Wallis test, Dunn’s *post*-*hoc* test). Spearman correlation between plant genes and bacterial OTUs in lateral roots (**B**). The triangles and dots indicated the bacterial OTUs and gene features respectively. The size of the triangles indicates the hub score. Red and blue solid lines indicate positive and negative correlations respectively. Only the hub OTUs connected with genes with significant plant gene ontology (GO) terms are labeled accordingly. Scatter plots illustrating the hub OTUs interacting with functional genes enriched in specific plant GO terms in the lateral root (**C**). Different color dots indicate different plant GO terms, referring to the network relationships. The size of the dots indicates the number of genes enriched in specific plant GO terms. Red and blue dots indicate positive and negative associations respectively. The actual length of the edge is measured as the Euclidean distance between the source node and the target node. **D** Relative abundance of *Massilia* among different mutants across the compartments. The size of the circles indicated the relative abundance (%). **E** Spearman correlation relationships between gene expression, OTU abundance and shoot biomass in the lateral root. The triangles, dots, and cubes indicated the microbiome, transcriptome, and biomass features respectively. Red and blue solid lines indicate positive and negative correlations respectively. The actual length of the edge is measured as the Euclidean distance between the source node and the target node. The size of the triangles indicates the hub score. Different color dots indicate specific plant gene ontology (GO) terms. The genus name of the hub OTUs and specific genes with GO annotation were labeled. *toc*, *timing of cab expression*; *shmt1*, *serine hydroxymethyltransferase 1*; *lnk*, *night light-inducible and clock-regulated*; *hsftf*, *heat stress transcription factor*

We also examined the 10 most abundant bacterial families for each compartment. Some families belonging to *Gemmatimonadaceae*, *Acidobacteriaceae*, *Geobacteraceae,* and *Planctomycetaceae* were most abundant in soil and gradually decreased from rhizosphere to root, cortex, and stele (Figure S8). The highly abundant families *Sphingomonadaceae* (RA = 0.07) and *Xanthomonadaceae* (RA = 0.04) were detected as the dominant taxa in the rhizosphere from the primary and lateral roots (Figure S8; Table S6). *Chitinophagaceae* were enriched in both root (RA = 0.20) and rhizosphere (RA = 0.18) (Figure S8). *Sinobacteraceae* and *Polyangiaceae* were specifically enriched in both lateral roots (RA = 0.06; RA = 0.07) and primary roots (RA = 0.05; RA = 0.07) (Fig. 3A). *Streptomycetaceae* were specifically enriched in lateral roots (RA = 0.11), which is significantly higher than the abundance in primary roots (RA = 0.07) and cortex (RA = 0.06) (Fig. 3A). Interestingly, *Comamonadaceae* (RA = 0.02) were highly enriched in all root tissues but not in soil, while *Burkholderiaceae* were highly enriched in cortex (RA = 0.45) and stele (RA = 0.83) as the dominant species (Figure S8; Table S6). In particular, *Oxalobacteraceae* were significantly enriched in primary roots (RA = 0.05), lateral roots (RA = 0.04), and cortex (RA = 0.08), and were also detected as a dominant taxon in the cortex (Fig. 3A, Table S6). These network associations and relative enrichment analyses demonstrate that different compartments recruit distinguished bacterial communities and that only specific bacteria inhabit distinct root tissues in maize. Collectively, *Oxalobacteraceae* is shown to be an important taxon and conserved in the lateral root and cortex tissue, thus highlighting its specific function linked with root developmental status and host plant gene expression.

### Keystone bacteria interact with host functional genes in the root

To construct potential associations between bacterial OTUs and host-expressed genes, we performed Spearman correlation analyses (rho > 0.7 or < −0.7 with FDR adjusted *P* < 0.05) for OTUs and genes expressed in ≥ 10 samples for root, cortex, and stele tissues. Among those, the lateral root rhizosphere had the most complex network with 1196 nodes and 3820 edges (Figures S9B and S10), while the primary root rhizosphere displayed the least complex network with only 544 nodes and 774 edges (Figures S9A and S10). We identified 39 hub OTUs from different compartments by selecting nodes that have a high hub score and where the associated genes have predicted functions. Among the hub OTUs, ~ 80% (31/39) of them are associated with the lateral root and its related rhizosphere compartment (Table S7). Interestingly, the highest connected OTU3535 (hub score = 1) is annotated to *Massilia* (*Oxalobacteraceae*) in the lateral root. We next explored the functional genes associated with hub OTUs and detected in total 19 unique gene ontology (GO) terms of those associated genes that have a positive or negative correlation with each hub OTU (Table S7). Among the GO terms in lateral roots, genes positively correlated to OTU3535 (*Massilia*) are enriched in “tryptophan biosynthetic process”, while negatively correlated genes have predicted function enriched in “rhythmic process” and “RNA biosynthetic processes” (Fig. 3B, C). We further checked the relative abundance of specific taxon *Massilia* across different compartments, and found that *Massilia* is highly abundant in the *lrt1* mutant, especially in the cortex tissue (Fig. 3D). These data indicate that keystone bacteria play an important role in interacting with functionally different host genes within the specified compartment.

### Keystone bacteria *Massilia* in lateral roots associated with plant phenology and flowering development

Overall, low nutrient treatment conditions significantly (one-way ANOVA, *P* < 0.05) reduced the shoot dry biomass (Figure S11A), while low nitrogen and low phosphorus treatment conditions significantly (one-way ANOVA, *P* < 0.05) reduced the nitrogen (Figure S11B) and phosphorus (Figure S11C) concentration, respectively. To identify whether keystone bacteria in association with host genes influence the maize phenotypes, we performed weighted correlation network analysis (WGCNA) between host genes and phenotypic traits as well as between bacteria and phenotypic traits across different nutrient treatment conditions and genotypes followed by Spearman correlation analysis between identified phenotypic traits related genes and bacteria (see “Materials and methods” section). We performed this integrative analysis using the data in the rhizosphere and root since we did not detect any significant correlations between host genes and bacteria in the cortex or stele tissue. In total, we identified different plant and microbial modules in association with plant traits, i.e., biomass and nutrient uptake (Figs. S12 and S13). We next focus on the genes from significant modules and detected the least complex network with only 19 nodes and 34 edges in the primary root rhizosphere (Figure S14A, Table S8), while the most significant correlations between host genes and bacteria related to shoot dry biomass in the rhizosphere of lateral roots with 243 nodes and 1202 edges (rho > 0.7 or < −0.7 and FDR < 0.05) (Figure S14B, Table S8). Moreover, there were more significant (rho > 0.7 or < −0.7 and FDR < 0.05) correlations between host genes and bacterial OTUs in the lateral roots than in the primary root (Figure S14C and Fig. 3E, Table S8). Interestingly, the hub OTU3535 (*Massilia*) in the lateral root significantly correlated with shoot dry biomass (*R*^2^_adj_ = 0.49, *P* = 0.0022, Figure S15) and had significant association with 103 genes expressed in lateral roots (Fig. 3E). We further examined the GO terms of these dry biomass-associated genes which are enriched in the GO term “circadian rhythm” (Fig. 3E). In particular, we observed the gene *night light-inducible and clock-regulated 1* (*lnk1*), which functions in response to abiotic stimulus and the gene *timing of cab expression 1* (*toc1*) which functions in flower development. For the primary root and its rhizosphere, functions of enriched GO terms were “iron transport” and “cell wall organization”, respectively (Figure S14A, C). Similarly, we performed network integration analyses for host genes, microbial OTUs, and shoot nitrogen and phosphorus concentration. We found only significant correlations between host gene expression and bacterial abundance related to nutrient uptake in the rhizosphere (Figure S16; Table S8). In the rhizosphere of primary roots, genes with GO functions in “cell wall organization” and “glucose metabolic process” had significant correlations with some keystone bacteria related to nitrogen concentration (Figure S16A), while in the rhizosphere of lateral roots, significantly enriched GO terms were, i.e., “phosphate starvation”, “phosphate transport” and “response to cold” for host genes, which significantly correlated with some keystone bacteria in association with nitrogen concentration (Figure S16B). We also observed phosphorus concentration-related genes with GO functions in “phosphate ion homeostasis” and “phosphate starvation” significantly correlated with keystone bacteria in the rhizosphere of lateral roots (Figure S16C). These integrative results may indicate that *Massilia* in lateral roots might influence maize biomass through associations with circadian rhythm and flowering development of host genes.

### Validation of *Massilia* function at different developmental stages in maize

**Fig. 4:**
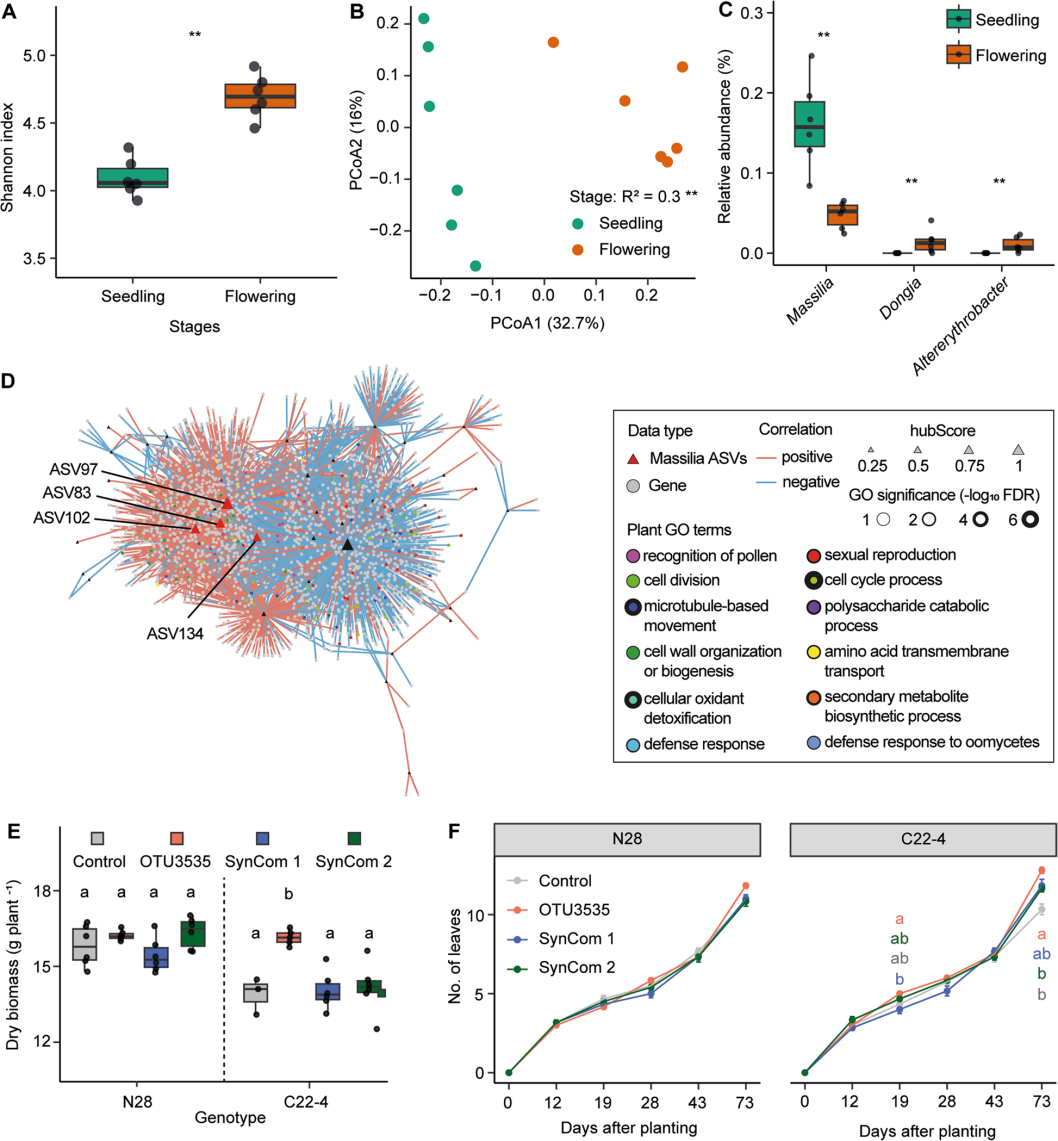
The bacterial hub *Massilia* associates with maize flowering and biomass production. **A** Root bacterial α-diversity (Shannon’s diversity index) at the seedling and flowering stage. Significances are indicated between different stages by an asterisk at *P* < 0.01 (two-tailed Wilcoxon rank-sum test). Boxes span from the first to the third quartiles, center lines represent median values and whiskers show data lying within the 1.5 × interquartile range of lower and upper quartiles. Data points at the ends of whiskers represent outliers. *n* = 6. **B** Principal coordinate analysis (PCoA) showing the dissimilarity of bacterial β-diversity between seedling and flowering stage. The explained variance (*R*^2^) by different stages was assessed by permutational analysis of variance (PERMANOVA, *P* < 0.01). *n* = 6. **C**, Significantly differential abundant genera between seeding and flowering stage. Significances were indicated among different stages by asterisk at *P* < 0.01 (two-tailed Wilcoxon rank-sum test). **D** Spearman correlation between high abundant ASVs and root differential genes between seedling and flowering stages. The triangles and dots indicated the bacterial OTUs and gene features respectively. Red and blue solid lines indicate positive and negative correlations respectively. The size of the triangles indicates the hub score. Different color dots indicate specific plant gene ontology (GO) terms. Effects of single inoculation of *Massilia* OTU3535 and Synthetic Community (SynCom) on the biomass production (**E**) and number of leaves (**F**) of maize wild-type N28 and its isogenic early flowering mutant C22-4. SynCom 1 is composed of 12 different bacterial isolates without OTU3535, while SynCom 2 is composed of 13 different bacterial isolates including OTU3535

The circadian rhythm in plants refers to physiologically relevant activity cycles of various biological processes regulated by an innate circadian clock, including growth, leaf development, and flowering transition. To explore whether *Massilia* is involved in maize flowering time, we performed a pot experiment using the same soil as described above for wild-type B73 and the *rtcs* mutant which contains both lateral roots and root hairs, and performed 16S amplicon (V3–V4) sequencing for the whole primary root with lateral roots at two different growth stages, i.e., at seedling stage (3 weeks) and flowering stage (10 weeks). Bacterial α-diversity analysis showed microbiome richness is significantly higher at the flowering stage compared to the seedling stage (*P* < 0.01, Wilcoxon rank-sum test) (Fig. 4A). In PCoA analysis, the developmental stage explained the large variance (*R*^2^ = 0.30, *P* < 0.01, PERMANOVA) of bacterial community composition (Fig. 4B). To investigate whether *Massilia* abundance changes during maize development, we compared the relative abundance for each dominant genus in the root of wild-type samples in control soil. In total, we detected three differentially abundant bacterial genera between stages, from which *Massilia* was the most abundant genus. Notably, its relative abundance significantly (*P* < 0.01, Wilcoxon rank-sum test) decreased from seedling to flowering stage (Fig. 4C). We next examined the root transcriptome and observed that the developmental stage effect can significantly separate the pattern of gene expression as shown by the PCA plot (Figure S16A). We determined differential gene expression between seedling and flowering stages and discovered that 2837 genes were differentially expressed between developmental stages (Figure S16B). To investigate whether some bacterial ASVs have significant associations with plant genes across developmental stages, Spearman correlation analysis was performed between highly abundant ASVs and differentially expressed genes. Interestingly, four hub ASVs (ASV83, ASV97, ASV102, and ASV134) all belonging to the bacterial genus *Massilia* were identified significantly in association with genes annotated in functional categories such as primary and secondary metabolic process, defense responses, and flowering-related pathways (Fig. 4D). We next further narrowed down the GO terms by removing the redundant ones and only highlight the driver GO terms (Table S9). Specifically, we identified two driver terms enriched in “recognition of pollen” and “detoxification” among the GO terms derived from ASV83 and ASV97 (Fig. 4D; Table S9). These integrated microbiome and transcriptome results suggest that hub *Massilia* might associated with plant genes involved in maize flowering.

### Growth promotion effect of *Massilia* depends on maize flowering time

To investigate whether the function of *Massilia* is associated with flowering time as a possible consequence of altered rhythmicity, we performed *Massilia* inoculation experiments for wild-type N28 and the corresponding early flowering mutant C22-4 in the greenhouse. We first performed alignment analysis using previously identified OTU3535 with these four hub ASVs and confirmed with > 97% sequence identity based on the BLAST alignment (Table S10). We then performed inoculation with the single OTU3535, the synthetic community with 12-member isolates derived from *Oxalobacteraceae* (SynCom 1), and 13-member isolates including OTU3535 (SynCom 2). Interestingly, only a single inoculation of *Massilia* OTU3535 significantly promoted maize dry biomass (Fig. 4E) and the number of leaves (Fig. 4F) in the early flowering mutant, whereas we did not observe any effects on these traits in the wild-type plants. Taken together, the function of *Massilia* in promoting maize biomass and leaf number might depend on flowering time in maize.

## Discussion

In this study, we systemically dissected different root organs and rhizosphere compartments (Fig. 1) along a single root at longitudinal and transversal resolution and subjected these samples to host-microbiome association by investigating the root transcriptome and associated microbiome using diverse maize root mutants. Examination of microbiomes across diverse mutants demonstrated that genotypes defective in lateral root formation displayed the largest changes in overall root gene expression and bacterial community composition in both the rhizosphere and the endosphere (Fig. 2). This is in agreement with earlier observations that lateral roots largely reshaped specific gene expression and microbial colonization in crops [37, 38]. Integrative trans-kingdom network association analyses demonstrated that the keystone bacterial taxon *Massilia* (*Oxalobacteraceae*) is highly associated with plant genes involved in plant circadian rhythm, tryptophan, and RNA biosynthetic processes in lateral roots (Fig. 3). Notably, in maize, the keystone bacterial taxon *Massilia* is significantly associated with the trait biomass and confers to a substantial enrichment of genes related to the circadian rhythm, e.g., flower development of plants (Fig. 3). Here, we demonstrated that lateral root specific recruitment of *Massilia* might contribute to the association with maize growth and the flowering time phenotype (Fig. 4), thus being the potential linkage between plant rhythmicity and variation of rhizosphere microbiome [39–41]. In the below sections, we discussed how our findings have deepened the understanding of root development and microbiome spatial assemblage in association with plant genetics and developmental biology.

### Spatial organization of root gene expression and rhizosphere microbiome assembly

Spatially organized pattern of root development coordinates plant gene expression and the microbiome assembly along the longitudinal root axis [36, 42, 43]. However, cereal root systems comprise diverse root types and specified organs such as lateral roots and root hairs supporting the high demand for water and nutrients of these plants in agricultural production [16, 19]. Nevertheless, little is known about how microorganisms assemble from soil to the rhizosphere and through specific root types or root tissues and what role such relatively abundant microorganisms play in crop performance. Previous transcriptomic and anatomical complexity of primary, seminal, and crown roots highlights root type-specific gene expression patterns and functional diversity in maize [44], meanwhile conferring diverse responses to environmental nutrients [19, 45] even with microbiome colonization such arbuscular mycorrhizal fungi [37, 38]. Our results demonstrated that gene expression showed a dynamic pattern from the primary root to the lateral root and different tissues such as the cortex and stele, which is in line with the different functions of root types and tissues [16]. The root system determines the main chemical messengers with associations with microorganisms in the root-soil interface, i.e., rhizosphere [19]. Thus, root type and tissue specification of gene expression could partially explain the synchronized pattern of assembled specific microbial taxa from the bulk soil through the rhizosphere and further to the roots and tissues. Our results are in line with plant selectiveness and recruitment of soil microbes into the host through separated rhizocompartments (rhizosphere, rhizoplane, and endosphere) [8, 46, 47], which is the critical step for initiation of such beneficial associations. Such synchronized patterns of root gene expression and microbial community assemblage could reflect the reciprocal interaction of lateral root development and specific enriched patterns of microbes [36, 48], suggesting that host gene expression drives the differentiation and specification of rhizosphere microbiome in crops. Understanding such co-assembled patterns of gene expression and microbial composition will help to understand how the microbiome adapted with host root systems in contribution to abiotic stress resilience, thus providing candidate genetic markers and gene regulatory components for breeding microbiome-driven crop fitness and stress resilience. Indeed, the plant-selected functional microbiome depends on the root exudation process that is released by the host [36, 49]. Future studies need to further explore the spatial pattern of root metabolic properties among root types and how such a fine-scale root exudation process will help to recruit specific functional microbial taxa in crops.

### Host genetics-encoded root mutation confers to larger effect on microbiome assembly than abiotic stresses

Understanding the microbiome assembly mechanism within a population and manipulation of functional microbiomes provides a promising strategy for breeding crop resilience and enhancing sustainable agriculture. Recent population genetic analyses support the hypothesis that plant genetic variation impacts microbiome assembly in crops [50–53]. Host-mediated microbial composition changes can have a large effect on plant performance [6], although the overall root microbiome is largely shaped by soil properties. In particular, such host-determined root development and microbial variation are largely influenced by abiotic stresses such as nutrient deficiency and drought [48, 54]. Especially, root morphological and anatomical features, e.g., lateral roots and root hairs play essential roles on overall absorption surface and beneficial plant-microbe interactions [19, 55–57]. Here, we found that lateral root mutation has a larger effect on microbiome assembly than root hair mutation. One potential explanation is that lateral root initiates from the deeply embedded pericycle cells in the stele tissue, and the formation of lateral roots will penetrate the whole cortical cell layers and epidermis, thus contributing to a large amount of root exudation to the rhizosphere [19]. Moreover, root hairs are tubular extensions of epidermal cells and significantly contribute to the increase of the absorbing surface of the root [16]. More importantly, the lateral root itself also initiates a substantial amount of root hairs, thus missing lateral roots will comprehensively reduce the overall root surface and biotic interactions with the soil microbiome. Recent studies highlight that shared molecular networks could have evolved through the interplay between rhizobia-mediated nodulation and lateral root development [58]. Indeed, the specific bacterial taxon *Massilia* has been found in driven lateral root development and host plant resistance to nitrogen stress by root-derived specific metabolites, i.e., flavonoids [36], and such associations between root and rhizosphere are genetically regulated [48]. These findings indicate that lateral root development mediated molecular component that guides root exudation processes may coordinate the engagement of host roots with microorganisms under nutrient deprivation. Finally, the abundance of *Massilia* is highly heritable in comparison to other bacterial taxa in the root under nitrogen-limited conditions [48], thus suggesting a great potential to breed stress-resilient crop root traits that more readily accommodate beneficial keystone bacteria.

### Community structure and function of specific microbiome depends on plant flowering time

Identification of the microbiome community and functional relationships with plant phenology is critical for determining the role of the plant microbiome in regulating plant growth and fitness. The composition and structure of plant-associated microbiomes are known to shift across seasons or developmental stages [7, 59, 60]. Interestingly, short timeframes such as specific circadian clock genes, i.e., *TIMING OF CAB EXPRESSION 1* (*TOC1*) and *LATE ELONGATED HYPOCOTYL* (*LHY*) had particularly pronounced impacts on rhizosphere community structure [39, 41]. In our network association analysis, we found that the functional bacterial taxon *Massilia* is significantly associated with several plant rhythmic genes including *TOC1* and *TOC2*. The potential explanation is the pervasive transcriptomic and phenotypic effects of clock misfunction on the plant host, thus potentially influencing the quantity and quality of root exudation-driven microbiome variation. Further studies demonstrated that some early- or late-flowering plants could select specific soil microbes in modification of plant traits [61], thus explaining the hypothesis plant microbiome could be associated with changes in different plant growth stages [59]. Surprisingly, our data indicated that *Massilia* is the most differential abundant bacterial taxon between seedling and flowering stage in maize. Together with published results, the beneficial function of the specific microbial taxon *Massilia* is developmental stage-dependent and involved in flowering time in maize.

Our study has several limitations. Firstly, our sampling specifically focused on the primary root of different mutants without consideration of other root types, e.g., the seminal root and crown roots, which indeed have different diversity metrics in maize [62]. Given the nature of different maize root types, i.e., root aging and developmental stages [16], it is likely that the microbiome assembly in the cortex and stele tissues may differ dramatically in comparison to the primary root. Nevertheless, we assume that the lateral root may recruit a similar pattern of specific microbes due to its biological conservation, i.e., initiation and elongation [19]. Future studies need to provide comprehensive information on plant gene expression and microbiome assembly along the whole root system and rhizosphere. Secondly, our study primarily applied the network approaches to identify the potential relationships among microbial OTUs or gene-OTU associations with plant traits. It should be noted that future work needs to dissect such associations using detailed genetic approaches, e.g., plant or microbial gene-encoded mutants, which were beyond the scope of this study. Thirdly, our study revealed that the function of *Massilia* depends on the early flowering mutant by inoculation experiment. However, it is important to note that flowering development is a very complex developmental process that involves a substantial number of plant genes. Further investigations are necessary to validate such findings of microbiome assembly using more genetic mutants in consideration of different developmental stages. Our future work will systemically elucidate the genetic basis and gene regulatory mechanisms of specific microbiome assembly in association with plant developmental processes.

## Conclusion

Taken together, our comprehensive transcriptome and microbiome analyses show that plant gene expression is associated with a spatial resolution of microbiome assembly from the soil through the rhizosphere to the root tissues in cereal maize. Overall the root system, lateral root mutation confers a larger influence on microbial assembly than root hair mutation. In particular, the abundance of bacterial taxon *Massilia* shows a specific developmental pattern and its beneficial effect is dependent on flowering time in maize. Our results provide important implications for the understanding of beneficial plant-microbial interactions in breeding new crop varieties with microbiome resilience.

## Materials and methods

### Maize mutants, soil preparation, and experimental design

Genetically distinct root mutants and wild-type B73 were used in this study. Among them, the *rtcs* mutant displays crown and seminal root defects; *rum1* displays seminal and lateral root defects; *lrt1* and *rth3-rth6* display lateral root and root hair defects [16]. C22-4 is a nearly isogenic line of wild-type N28. C22-4 carries Gaspe Flint introgressions on Chromosome 8 and shows an early flowering phenotype [63]. We used the soil that was dug from a long-term (over 100 years) fertilizer field experiment in Dikopshof (50˚48′21′′N, 6˚59′9′′E). We have collected three different soils e. g. the control soil with fully fertilized nutrients (CK), low nitrogen soil fertilized without nitrogen (LN), and low phosphorus soil fertilized without phosphorus (LP). The detailed soil nutrient information was described by He et al. (2024). The freshly dug soil was air-dried and sieved through a 4-mm sieve ready for use. The soil pot (7 cm × 7 cm × 19 cm) experiment was carried out in a complete randomized design and comprised seven genotypes and three nutrient treatment conditions. In total, four biological replicates were performed. We prepared additional empty soil pots without plants grown as the “bulk soil” samples. All pots in each culture tray were completely randomized using a true random generator (excel function “RAND”) and trays were reshuffled every week in the growth chamber without paying attention to the pot labels. Specifically, we maintained the soil water content by weighing the soil pots according to the loss of water every 2 days. For the whole 4-week culture, only sterilized water was applied to avoid potential contamination.

### Sample collection and tissue separation

The root and rhizosphere samples were harvested from 4-week-old maize plants for all genotypes grown under different nutrient treatment conditions. In detail, the whole root systems were carefully taken out from each pot and vigorously shaken to remove all soil not firmly attached to the roots. To synchronize the harvest for precise comparison among different maize mutants, we specifically focused on the primary root which is present in all mutants. We separately dissected the lateral roots from the primary root as previously described [38]. Moreover, we carefully removed all big particles from these primary or lateral roots to avoid contamination. Only the root organs with tightly attached soil were placed into a 15-ml Falcon (Sarstedt) tube and immediately frozen in liquid nitrogen and stored at −80 °C before extraction of rhizosphere soil. The rhizosphere samples were defined and extracted into PowerBead tubes (MP Biomedical) as described previously [36]. The root samples were harvested from a separate plant and the attached soil was washed immediately with tap water and rinsed with three times sterilized water followed by tissue drying and placed in PowerBead tubes. Moreover, the stele and cortex tissue from the differentiation zone of the primary root was peeled off separately by hand as previously described [45]. Please note that we were not able to separate these two tissues in the zone with emerged lateral roots. We collected the bulk soil samples from the unplanted pots as the control.

### RNA sequencing and bacterial 16S rRNA gene sequencing

The frozen rhizosphere samples were extracted from the primary root and lateral roots respectively as described previously [36]. DNA extractions were performed soon after root and rhizosphere samples were harvested, following the FastDNA™ SPIN Kit for Soil (MP Biomedical) protocol. Total RNA was isolated from the primary root, lateral root, cortex, and stele tissue samples using the RNeasy Plus Universal Mini Kit (Qiagen). Both DNA and RNA were qualified and quantified via Agilent RNA or DNA Chips (Agilent Technologies). The complementary DNA libraries for RNA-seq were constructed with the MGIEasy RNA library construction kit. Cluster preparation and PE150 read sequencing were performed on a DNBSEQ-G400 system. Amplicon library construction was processed with a similar workflow as previously described [36]. In brief, the full length (V1–V9 region) of the 16S rRNA genes was sequenced on a Pacbio Sequel II (PacBio Biosciences Inc.) using the forward primer (27F) with anchor sequence 5′-TTTCTGTTGGTGCTGATATTGCAGRGTTYGATYMTGGCTCAG-3′ and reverse primer (1492R) with anchor sequence 5′-ACTTGCCTGTCGCTCTATCTTCCGRGYTACCTTGTTACGACTT-3′ [64].

### Transcriptome data analysis

Processing and trimming of raw RNA-seq reads were performed as described previously [36]. In brief, the low-quality sequences and low-complexity polyA tails were eliminated. Subsequently, we built the reference genome index and mapped the sequences to the maize reference genome sequence v.5 (http://ftp.ensemblgenomes.org/pub/plants/current/fasta/zea_mays/dna/Zea_mays.Zm-B73-REFERENCE-NAM-5.0.dna.toplevel.fa.gz) by HISAT2 (v2.1.0) software [65]. All commands and default parameters were used in HISAT2. All downstream analyses were performed in R (v4.2.2) [66]. Then all bam files generated by HISAT2 were input to ‘featureCounts’ function [67] in R package Rsubread (v2.12.3) using maize reference annotation v.5 (http://ftp.ensemblgenomes.org/pub/plants/current/gtf/zea_mays/Zea_mays.Zm-B73-REFERENCE-NAM-5.0.53.chr.gtf.gz) to generate the gene expression table. Chimeric reads and reads mapped to more than one position in the genome were removed. Only genes represented by a minimum of ten mapped reads in ≥ 4 samples were declared as expressed and considered for downstream analyses. Before statistical analyses, data were normalized by library size using the DESeq2 (v1.38.3) package [68] in R. A principal component analysis was performed using the ‘prcomp’ function in base R (v4.2.2). To test the marginal effects of compartment, nutrient treatment conditions, and genotype on the transcriptome, a permutation-based PERMANOVA test was performed with the Euclidean distance matrix between pairs of transcriptomic samples using ‘adonis2’ function in R package vegan (v2.6.4) [69]. All plots were produced using R package ggplot2 (v3.4.2) [70].

### Bacteria whole 16S rRNA gene sequence processing and data analysis

16S rRNA gene (V1–V9) raw sequencing reads were processed as following steps. Sequencing reads were assigned to samples based on their unique barcode and truncated by cutting off the barcode and primer sequence which were called raw tags. Raw sequence data were quality filtered and deduplicated using Usearch fastx_uniques command. OTUs (Operational taxonomic units) were generated by the UPARSE [71] algorithm in the Usearch software (v11.0.667) with parameters for full-length sequences. Sequences were clustered based on 97% identity and assigned to a different OTU using Usearch cluster_otus. Taxonomy was assigned to OTUs using the BLCA software (v2.2) [72] against the NCBI 99% OTUs reference sequences (20170709) at each taxonomic rank (kingdom, phylum, class, order, family, genus, species). During the clustering process, the chimeric sequences were removed using UCHIME to filter the final OTU sequences using the RDP “gold” sequences [73]. Mitochondria, chloroplast, and phylum-unassigned OTUs were eliminated.

All downstream statistical analyses were performed in R (v4.2.2) (R Core Team 2022). Briefly, OTU tables were filtered with ≥ 0.1% relative abundance in ≥ 2 samples. For α-diversity indices, the Shannon index was calculated using OTU tables rarefied to 10,000 reads. The abundant OTUs that expressed ≥ 0.1% relative abundance in ≥ 5% samples were kept and no samples were removed. Bray–Curtis distances between samples were calculated using OTU tables that were normalized using ‘varianceStabilizingTransformation’ function from DESeq2 (v1.38.3) package [68] in R. If not specified, the following data analysis is based on the normalized OTU table. Principal coordinate analyses were performed using the ‘ordinate’ function in R package phyloseq (v1.42.0) [74]. To test the marginal effects of compartment, nutrient treatment conditions, and genotype on the microbial composition community, a permutation-based PERMANOVA test was performed using the Bray–Curtis distance matrix between pairs of bacterial samples using ‘adonis2’ function in R package vegan (v2.6.4) [69]. All plots were produced using R package ggplot2 (v3.4.2) [70].

### Differential gene expression and functional characterization

We determined differential gene expression between each mutant and wild type (B73) using the ‘DESeq’ function in DESeq2 R package (v1.38.3). Subsequently, we determined the number of differentially expressed genes for each comparison by controlling the FDR-adjusted *P* values of pairwise *t*-tests to 0.05 and a fold change of > 2. We then functionally classified differential gene expression patterns according to GO terms using g:Profiler [75]. The GO annotation system is based on four structured vocabularies that describe gene products in terms of their associated biological processes, cellular components, molecular functions, and KEGG pathways in a species-independent manner. Subsequently, we performed a gene set enrichment analysis to discover significantly overrepresented functional categories.

### Functional prediction of bacterial OTUs

PICRUSt2 (v2.5.2) [76] was used to predict functional pathways present in bacterial communities from the 16S rRNA marker gene. KEGG orthology (KO) abundance table was predicted through the PICRUSt2 pipeline with the input of OTU-table and OTUs sequences. To keep only abundant KOs, KOs that express > 0.01% relative abundance in > 5% samples were kept. After filtering, individual KOs were summarized at KEGG pathway level 3 using the categorize_by_function_l3 function and KEGG mapping file provided by PICRUSt group. KEGG pathway level 2 and level 1 were manually curated from the KEGG website (https://www.genome.jp/kegg). Then we identified differential KEGG pathways between each mutant and wild type (B73) using the ‘DESeq’ function in DESeq2 R package (v1.38.3). Subsequently, we determined the number of differential abundance pathways for each comparison by controlling the FDR-adjusted *P* values of pairwise *t*-tests to 0.05 and a log_2_ fold change of > 0.5.

### Construction of bacterial cooccurrence networks

To explore the potential associations among different bacterial OTUs, we constructed bacterial cooccurrence networks within each compartment using the highly abundant OTUs > 0.1% in at least 10% of samples. These filtered OTUs were used as the input for the SparCC algorithm [77], which referred to the compositional data, i.e., relative fractions of species or OTUs, rather than their absolute abundances. This analysis was performed with default parameters and 100 bootstraps were used to infer *P* values. The correlations > 0.4 or < −0.4 (*P* < 0.05) were kept for network construction. Networks were visualized using spring embedded layout with weight in Cytoscape (v3.8.0) [78].

### Trans-kingdom associations between root genes and bacterial OTUs

To get the associations between plant genes and microbial OTUs, we decided to use Spearman’s rank correlation coefficient, i.e., a nonparametric measure of rank correlation, which can well evaluate the relationship between two variables and can be described using a monotonic function. In this case, gene expression data and microbial relative abundance can be ranked separately with a certain order, and then association was produced independently of the data types, i.e., root expressed gene expression and root/rhizosphere abundant OTUs. To reduce false positive correlations, only genes expressed > 5 reads in at least 10 samples were used as the gene input table, and only OTUs expressed in at least 10 samples were kept for the OTU input table. After normalization using ‘varianceStabilizingTransformation’ function from DESeq2 package for both the gene table and OTU table, the Spearman correlation was calculated by ‘corr.test’ function from psych package (v2.3.3) [79] in R for each compartment. This function provides the correlation coefficient and the corresponding FDR-adjusted *P* values. Spearman correlations with rho value > 0.7 or < −0.7 and adjusted *P* values < 0.05 were kept as significant correlations. The above significant correlations were input to Cytoscape (v3.8.0) [78] for network visualization. Node hub scores were calculated using the ‘hub_score’ function from igraph package (v1.4.2) [80] in R.

### Integration of host transcriptome, bacterial community, and phenotypic traits

Network-based analysis is the most biologically interpretable tool available to analyze association among variables, such as relationships between microbial compositions, gene expression, and phenotypic traits [81]. Weighted correlation network analysis (WGCNA) is a data-driven method that clusters genes into different modules based on weighted correlations between gene transcripts [82]. To identify keystone bacteria that significantly associate with phenotypic traits and also with host transcriptome in each compartment, we performed WGCNA for both bacteria OTUs and host root expressed genes in R: (1) cluster gene/OTU co-expression modules among different genotypes across three nutrient conditions. (2) Associate module eigengene/eigenOTU with phenotypic traits. (3) Select genes/OTUs with high membership value from modules that are significantly associated with phenotypic traits and high correlation coefficient with phenotypic traits. (4) Correlate the selected genes and OTUs to determine key OTUs and genes.

For robust construction of co-expression networks, we filtered and normalized the genes/OTUs table as described above. The soft thresholding power β was automatically selected and used to calculate the adjacency matrix. To minimize the effects of noise and false associations, we transformed the adjacency matrix into a topological overlap matrix (TOM) with selected power and calculated the corresponding dissimilarities (dissTOM) as 1–TOM. For hierarchical clustering of genes/OTUs we used dissTOM as a distance measure and set the minimum module size (number of genes) to 30 (number of OTUs to 3) to detect modules. To quantify the co-expression similarities of entire modules, their eigengenes/eigenOTUs were calculated and subsequently used to associate with phenotypic traits. We chose modules with *P* values < 0.05 as significantly associated modules. Then we calculated the Spearman correlation between normalized genes/OTUs expression and phenotypic traits as well as gene/OTU membership value using ‘signedKME’ function from the WGCNA package in the above selected significant modules. The key genes/OTUs were determined by selecting the overlapping genes/OTUs between genes/OTUs which have Spearman correlation coefficients > 0.7 or < −0.7 and *P* values < 0.05 and genes/OTUs which have membership value > 0.7 or < −0.7. Then we did Spearman correlation between the key genes and OTUs to find significant associations by selecting *rho* > 0.7 or < −0.7 and FDR adjusted *P* values < 0.05. Network visualization was performed in Cytoscape as described above. For each keystone OTU, we functionally classified the interacted root genes that enriched into different GO terms using g:Profiler [75].

### Validation experiment 1: comparison of different growth stages

To verify if *Massilia* is associated with maize developmental stages, we grew wild-type B73 in control soil and harvested their root samples at the seedling stage and flowering stage. RNA and DNA extractions were performed as described above. We then performed the RNAseq and 16S rRNA (V3–V4) gene sequencing for the root samples. The amplicon libraries were sequenced at the V3-V4 regions of the 16S rRNA gene were amplified using a 16S (V3–V4) Metagenomic Library Construction Kit for NGS (Takara Bio Inc., Kusatsu, Japan). The following primers were used: 341F with overhang adapter 5′-TCGTCGGCAGCGTCAGATGTGTATAAGAGACAGCCTACGGGNGGCWGCAG-3′ and 806R with overhang adapter 5′-GTCTCGTGGGCTCGGAGATGTGTATAAGAGACAGGGACTACHVGGGTWTCTAAT-3′. The second PCR was performed using the Nextera® XT Index Kit (Illumina, San Diego, CA, USA) for sample multiplexing with index adapters. The libraries were sequenced on the MiSeq™ platform using the MiSeq™ Reagent Kit v3 (2 × 250 bp; Illumina). Raw reads were processed following a similar workflow as previously described [36]. Briefly, paired-end 16S rRNA amplicon sequencing reads were assigned to samples based on their unique barcode and truncated by cutting off the barcode and primer sequence. Sequence analyses were performed by QIIME 2 software (v2020.6) [83]. Raw sequence data were demultiplexed and quality filtered using the q2‐demux plugin followed by denoising with DADA2 [84] (via q2‐dada2). Sequences were truncated at position 250 and each unique sequence was assigned to a different ASV. Taxonomy was assigned to ASVs using the q2‐feature‐classifier [85] and the classify‐sklearn naïve Bayes taxonomy classifier against the SSUrRNA SILVA 99% OTUs reference sequences (v138) [86] at each taxonomic rank (kingdom, phylum, class, order, family, genus, species). Mitochondria- and chloroplast-assigned ASVs were eliminated. Out of the remaining sequences (only ASVs with ≥ 0.05% relative abundance in ≥ 2 samples) were kept to build an ASV table.

All downstream analyses were performed in R (v4.2.2) [66]. For α-diversity indices, the Shannon index was calculated using the ASV table rarefied to 1000 reads. For all following analyses, abundant ASVs were used, so ASVs that express ≥ 0.05% relative abundance within ≥ 5% samples were kept. After filtering taxa, the samples with ≤ 1000 reads were also removed. Bray-Curtis distances between samples were calculated using the ASV table that was normalized using the ‘varianceStabilizingTransformation’ function from DESeq2 (v1.38.3) package [68] in R. If not specified, the following data analysis is based on the normalized ASV table. Principal coordinate analyses were performed using the ‘ordinate’ function in R package phyloseq (v1.42.0) [74]. To test the marginal effects of nutrient treatment conditions and genotype on the microbial composition community, a permutation-based PERMANOVA test was performed using Bray–Curtis distance matrix between pairs of bacterial samples using ‘adonis2’ function in R package vegan (v2.6.4) [69]. To identify genera differentially expressed between stages, ASVs were grouped by genus, and unidentified genera were removed. Only genera that express > 0.1% in ≥ 2 samples were kept. Then relative abundance of each genus was compared between stages using Wilcoxon rank sum test [87]. All plots were produced using R package ggplot2 (v3.4.2) [70]. In the end, we performed similar Spearman correlation analyses using the differentially expressed genes and differentially abundant OTUs for the seedling and flowering stage. Network visualization, definition of keystone OTU, and functional classification of GO terms were performed as described above.

### Validation experiment 2: *Massilia* inoculation in early flowering maize mutant

To explore the potential effects of *Massilia* on plant performance, we carried out a growth promotion assay for wild-type N28 and its early flowering time mutant C22-4 by inoculation with different *Massilia* strains (He et al. 2024). In detail, the bacterial strains were isolated using R2A (CARL ROTH) media supplemented with 100 µg mL^−1^ Cyclohexamid (CARL ROTH) from the rhizosphere or root of 4-week-old maize plants in the soil pot experiment described above. The isolates were randomly picked out from the plates with colony-forming units (CFUs) ranging between 30 and 100 CFUs. From all isolates, we identified several isolates which were aligned to *Massilia*. Before the inoculation experiment, we first aligned the sequences of different *Massilia* strains to the 16S (V1–V9) sequence of the hub OTU3535 using BLASTn (v2.6.0) [88] with default parameters and we chose the 100% alignment isolate as the candidate used in the following inoculation experiment. We applied three different inoculation strategies, i.e., single inoculation using isolate which has 100% identity with *Massilia* OTU3535 (SynCom1), 12 isolates belonging to Oxalobacteraceae excluding *Massilia,* and all 13 isolates including *Massilia* (SynCom2) in nitrogen-poor soil. We used the same soil as in the previous soil pot experiment and sterilized it according to an established protocol [36]. Seed sterilization, isolate preparation, root inoculation, and growth assay were performed as previously reported [48]. The wild-type plants and mutants were grown in the greenhouse (16/8 h light/dark and 26/18 °C) for 1 month. Then plants were harvested, and the shoot-dry biomass and number of fully developed leaves were determined.

### Supplementary Information

Supplementary Material 1: Supplementary Figure S1. Overlapped genes (A) and OTUs (B) among all compartments. Rh_PR, Rhizosphere from primary roots; Rh_LR, Rhizosphere from lateral roots; PR, Primary roots; LR, Lateral roots. Expressed genes are defined as reads >5 in at least 4 samples. Expressed OTUs are defined as relative abundance >0.1% in at least 2 samples. Supplementary Figure S2. Bacterial α-diversity among genotypes (A) and treatments (B) across rhizosphere and root compartments. α-diversity was estimated by Shannon’s diversity index. Compartment significances were calculated using a Kruskal-Wallis test with post-hoc Dunn’s test (Benjamini-Hochberg adjusted *P* <0.05). Different letters indicate significance among different genotypes or treatments (Benjamini-Hochberg adjusted *P* <0.05). Rh_PR, Rhizosphere from primary root; Rh_LR, Rhizosphere from lateral root; PR, Primary root; LR, Lateral root. *rum1*, *rootless with undetectable meristem 1*; *rtcs*, *rootless concerning crown and seminal roots*; *lrt1*, *lateral rootless 1*; *rth*, *roothairless*. B73 is the wild type plant. Boxes span from the first to the third quartiles, center lines represent median values and whiskers show data lying within 1.5× interquartile range of lower and upper quartiles. Data points at the ends of whiskers represent outliers. Supplementary Figure S3. Principal component analysis (PCA) illustrating the transcriptomic dissimilarity among genotypes and treatments for each compartment. A, Primary root; B, Lateral root; C, Cortex tissue; D, Stele. *rum1*, *rootless with undetectable meristem 1*; *rtcs*, *rootless concerning crown and seminal roots*; *lrt1*, *lateral rootless 1*; *rth*, *roothairless*. The explained variance by genotype, nutrient treatment condition and interaction were assessed by permutational analysis of variance (PERMANOVA, *P* <0.001). Supplementary Figure S4. Principal coordinate analysis (PCoA) showing the dissimilarity of bacterial β-diversity for each compartment. A, Rhizosphere from primary root; B, Rhizosphere from lateral root; C, Primary root; D, Lateral root; E, Cortex tissue; F, Stele. *rum1*, *rootless with undetectable meristem 1*; *rtcs*, *rootless concerning crown and seminal roots*; *lrt1*, *lateral rootless 1*; *rth*, *roothairless*. The explained variance by genotype, nutrient treatment condition and interaction were assessed by permutational analysis of variance (PERMANOVA, *P* <0.001). Supplementary Figure S5. PERMANOVA results for PCoA of bacterial community composition and PCA of gene expression. Rh_PR, Rhizosphere from primary root; Rh_LR, Rhizosphere from lateral root; PR, Primary root; LR, Lateral root. Supplementary Figure S6 OTU-OTU co-occurrence network in soil (A), rhizosphere of primary root (B), rhizosphere of lateral root (C), primary root (D), lateral roots (E), cortex (F) and stele (G). Nodes color represents phylum, node size is proportional to hub score and node border width is proportional to mean relative abundance. Key OTUs are labeled by OTU id. Red and blue solid lines indicate positive and negative correlations respectively. Supplementary Figure S7. Number of nodes and edges of OTU-OTU SparCC network within each compartment. Rh_PR, Rhizosphere from primary root; Rh_LR, Rhizosphere from lateral root; PR, Primary root; LR, Lateral root. Supplementary Figure S8. Relative abundance of top ten rich families across different compartments. Rh_PR, Rhizosphere from primary root; Rh_LR, Rhizosphere from lateral root; PR, Primary root; LR, Lateral root. Significances were indicated among different compartments by different letters for each family (Benjamini-Hochberg adjusted *P* < 0.05, Kruskal-Wallis test, Dunn’s *post*-*hoc* test). Supplementary Figure S9. Network associations between plant genes and microbial OTUs in the rhizosphere from primary root (A) and lateral root (B). The triangles and dots indicated the bacterial OTUs and gene features respectively. The size of the triangles indicates the hub score. Red and blue solid lines indicate positive and negative correlations respectively. Only the hub OTUs connected with genes with significant plant gene ontology (GO) terms are labelled accordingly. Supplementary Figure S10. Number of edges, genes, and OTUs for each OTU-Gene network. Rh_PR, Rhizosphere from primary root; Rh_LR, Rhizosphere from lateral root; PR, Primary root; LR, Lateral root. Supplementary Figure S11. Maize phenotypic traits under different treatments. A, Shoot dry biomass; B, Nitrogen concentration; C, Phosphorus concentration. N, nitrogen; P, phosphorus. Samples are colored in genotypes. Different letters indicate significant differences controlled by One-Way ANOVA at the *P* <0.05. Supplementary Figure S12. Gene WGCNA modules and their correlations with plant traits. A, Primary root; B, Lateral root. Supplementary Figure S13. Bacterial WGCNA modules and their correlations with plant traits. A, Rhizosphere from primary root; B, Rhizosphere from lateral root; C, Primary root; D, Lateral root. Supplementary Figure S14. Bacterial hubs prioritize the causal association with plant rhythmic process and biomass accumulation. Spearman correlation relationships between gene expression, OTU abundance and shoot biomass in the rhizosphere from primary root (A), rhizosphere from lateral root (B) and primary root (C). The triangles, dots and cubes indicated the microbiome, transcriptome and biomass features respectively. Red and blue solid lines indicate positive and negative correlations respectively. The actual length of the edge measured as the Euclidean distance between the source node and the target node. The size of the triangles indicates the hub score. Different color dots indicate specific plant gene ontology (GO) terms. The genus name of the hub OTUs and specific genes with GO annotation were labelled. Supplementary Figure S15. linear model between shoot dry biomass and OTU3535 relative abundance (%). Linear model was fitted using lm() function. Supplementary Figure S16. Trans-kingdom interaction network between bacterial OTUs and root genes in association with plant nutrients concentration. A, Root gene expression, bacterial OTUs in the rhizosphere from primary root and nitrogen concentration; B, Root gene expression, bacterial OTUs in the rhizosphere from lateral root and nitrogen concentration; C, Root gene expression, bacterial OTUs in the rhizosphere from lateral root and phosphorus concentration. Supplementary Figure S17. PCA plot and differential expression genes between flowering stage and seedling stage. PERMANOVA test was performed to calculate the variance explained by stage in gene expression (permutations = 1999). Differential expression genes were determined by set absolute value of log_2_Foldchange >2 and FDR adjusted *P* <0.01.

Supplementary Material 2: Supplementary Table S1. Bacteria OTU table consisting of 1098 OTUs from 388 samples. Supplementary Table S2. PERMANOVA results for bacteria microbiota and root RNA sequencing. Supplementary Table S3. Detailed list of functional predicted microbial metabolism pathways using PICRUSt tool. Supplementary Table S4. Significant enriched KEGG pathways of differential expression genes between lateral root mutant and wild type B73. Supplementary Table S5. Top hub score OTUs in each OTU-OTU SparCC network. Supplementary Table S6. Indicator families in each compartment. Supplementary Table S7. Hub OTUs and GO functions of significantly correlated genes with each hub OTU in each gene-OTU network. Supplementary Table S8. Network associations between genes and OTUs underlying plant traits. Supplementary Table S9. Detailed list of GO terms of DE genes significantly correlated with ASVs. Supplementary Table S10. Sequence alighment results using BLASTn between OTU3535 and ASVs detected in validation experiment.

## Data Availability

All raw plant RNA-seq data, bacterial 16S sequencing data reported in this paper were deposited in the Sequence Read Archive (http://www.ncbi.nlm.nih.gov/sra) under accession no. PRJNA1018308. RNA-seq reads were mapped to the maize reference genome sequence v.5 (http://ftp.ensemblgenomes.org/pub/plants/current/fasta/zea_mays/dna/Zea_mays.Zm-B73-REFERENCE-NAM-5.0.dna.toplevel.fa.gz) and were annotated based on the reference gene models v.5 (http://ftp.ensemblgenomes.org/pub/plants/current/gtf/zea_mays/Zea_mays.Zm-B73-REFERENCE-NAM-5.0.53.chr.gtf.gz). The GO terms were annotated using g:Profiler (https://biit.cs.ut.ee/gprofiler/gost). We deposited all raw OTU table, OTU taxonomy, gene table and customized scripts including all downstream analysis and the association of gene, bacteria and phenotypic traits in the following GitHub repository:https://github.com/Danning16/Integrated-analysis-of-bacteria-and-transcriptome-along-the-primary-root-in-maize.
