# Supplementary material for: Lateral root enriched Massilia associated with plant flowering in maize
Source: Microbiome. 2024 Jul 9;12:124. doi: 10.1186/s40168-024-01839-4 (PMC11234754; doi:10.1186/s40168-024-01839-4)
Supplement: Supplementary file 2 — Supplementary Material 1: Supplementary Figure S1. Overlapped genes (A) and OTUs (B) among all compartments. Rh_PR, Rhizosphere from primary roots; Rh_LR, Rhizosphere from lateral roots; PR, Primary roots; LR, Lateral roots. Expressed genes are defined as reads >5 in at least 4 samples. Expressed OTUs are defined as relative abundance >0.1% in at least 2 samples. Supplementary Figure S2. Bacterial α-diversity among genotypes (A) and treatments (B) across rhizosphere and root compartments. α-diversity was estimated by Shannon’s diversity index. Compartment significances were calculated using a Kruskal-Wallis test with post-hoc Dunn’s test (Benjamini-Hochberg adjusted P <0.05). Different letters indicate significance among different genotypes or treatments (Benjamini-Hochberg adjusted P <0.05). Rh_PR, Rhizosphere from primary root; Rh_LR, Rhizosphere from lateral root; PR, Primary root; LR, Lateral root. rum1, rootless with undetectable meristem 1; rtcs, rootless concerning crown and seminal roots; lrt1, lateral rootless 1; rth, roothairless. B73 is the wild type plant. Boxes span from the first to the third quartiles, center lines represent median values and whiskers show data lying within 1.5× interquartile range of lower and upper quartiles. Data points at the ends of whiskers represent outliers. Supplementary Figure S3. Principal component analysis (PCA) illustrating the transcriptomic dissimilarity among genotypes and treatments for each compartment. A, Primary root; B, Lateral root; C, Cortex tissue; D, Stele. rum1, rootless with undetectable meristem 1; rtcs, rootless concerning crown and seminal roots; lrt1, lateral rootless 1; rth, roothairless. The explained variance by genotype, nutrient treatment condition and interaction were assessed by permutational analysis of variance (PERMANOVA, P <0.001). Supplementary Figure S4. Principal coordinate analysis (PCoA) showing the dissimilarity of bacterial β-diversity for each compartment. A, Rhizosphere from primary [file 40168_2024_1839_MOESM1_ESM.docx]

**Supplemental files**

Supplemental figures 1-17


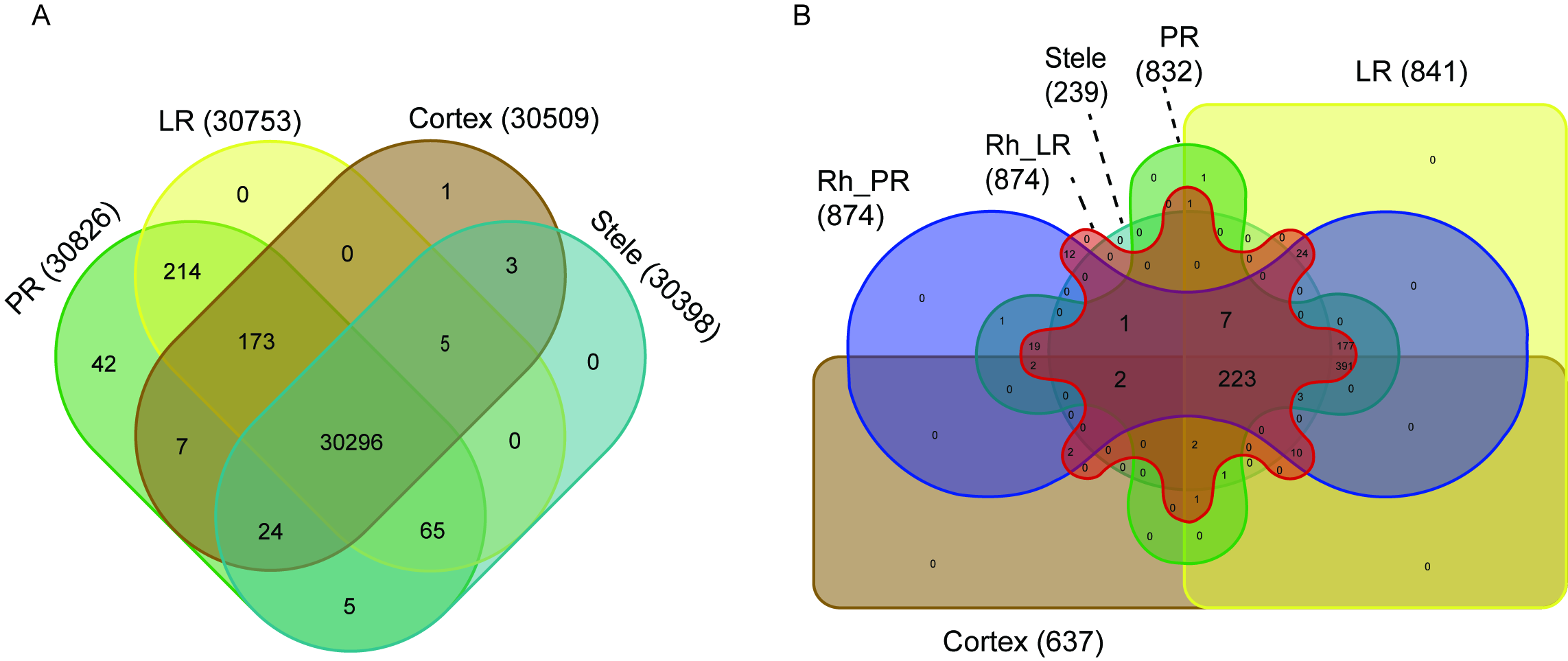


**Fig. S1. Overlapped genes (A) and OTUs (B) among all compartments.** Rh_PR, Rhizosphere from primary roots; Rh_LR, Rhizosphere from lateral roots; PR, Primary roots; LR, Lateral roots. Expressed genes are defined as reads >5 in at least 4 samples. Expressed OTUs are defined as relative abundance >0.1% in at least 2 samples.


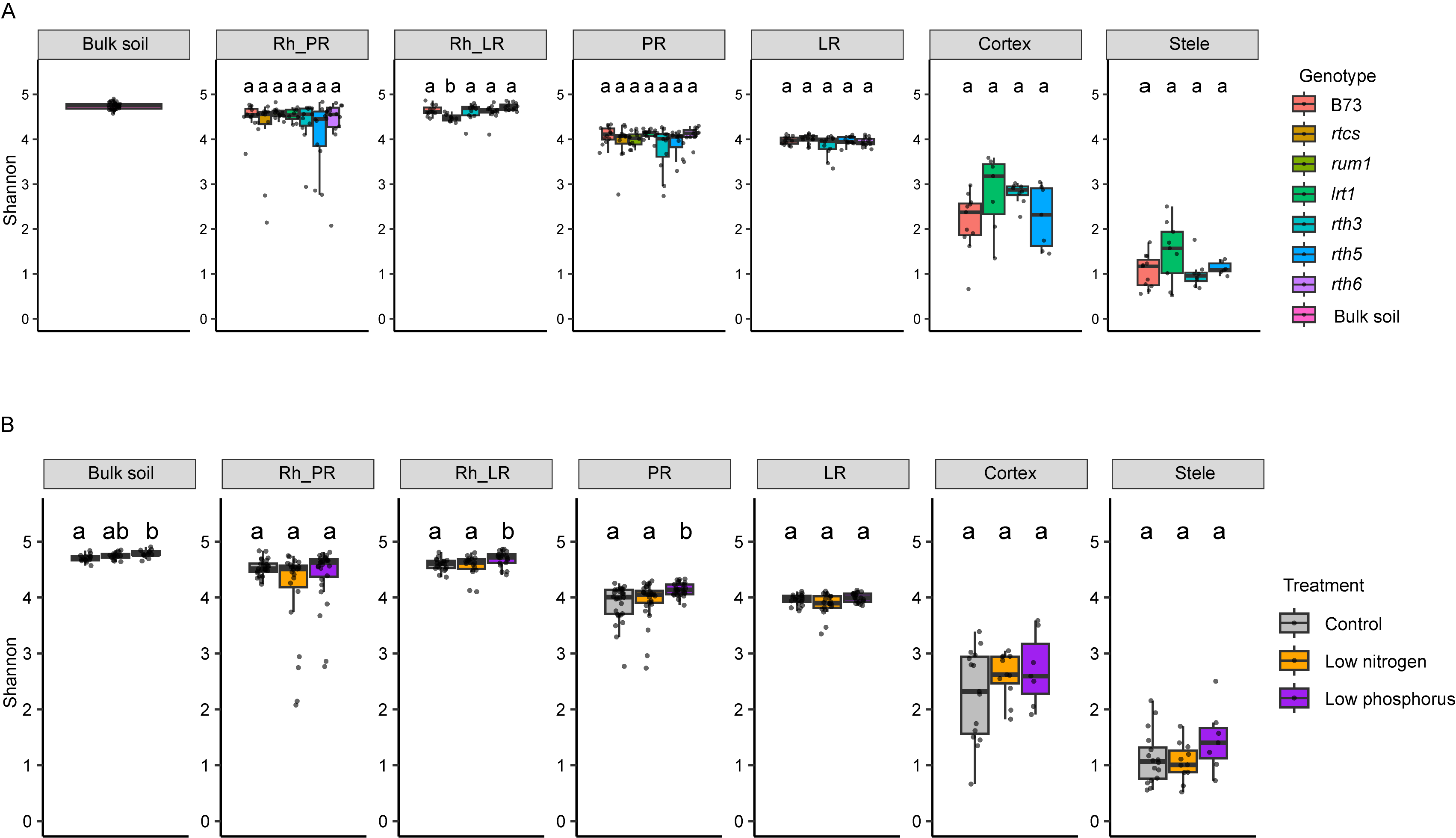


**Fig. S2. Bacterial α-diversity among genotypes (A) and treatments (B) across rhizosphere and root compartments.** α-diversity was estimated by Shannon’s diversity index. Compartment significances were calculated using a Kruskal-Wallis test with post-hoc Dunn’s test (Benjamini-Hochberg adjusted *P* <0.05). Different letters indicate significance among different genotypes or treatments (Benjamini-Hochberg adjusted *P* <0.05). Rh_PR, Rhizosphere from primary root; Rh_LR, Rhizosphere from lateral root; PR, Primary root; LR, Lateral root. *rum1*, *rootless with undetectable meristem 1*; *rtcs*, *rootless concerning crown and seminal roots*; *lrt1*, *lateral rootless 1*; *rth*, *roothairless*. B73 is the wild type plant. Boxes span from the first to the third quartiles, centre lines represent median values and whiskers show data lying within 1.5× interquartile range of lower and upper quartiles. Data points at the ends of whiskers represent outliers.


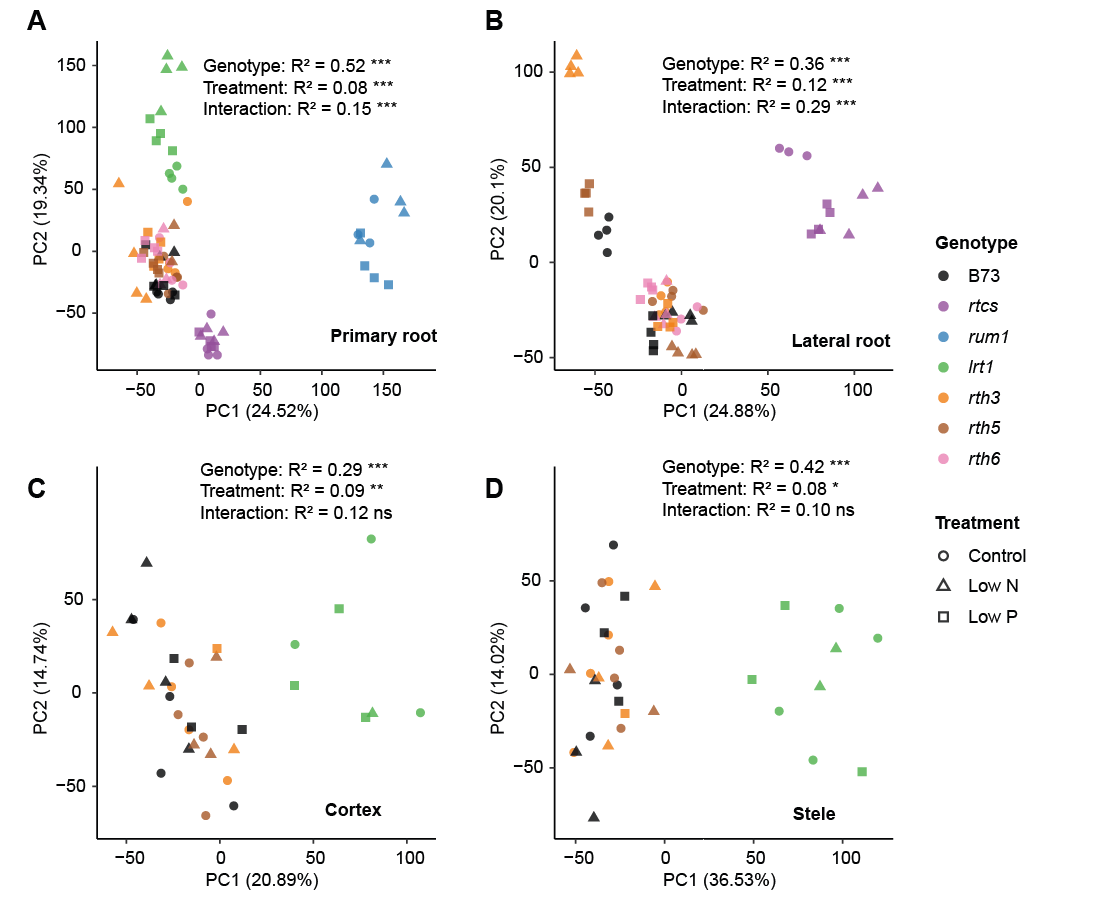


**Fig. S3. Principal component analysis (PCA) illustrating the transcriptomic dissimilarity among genotypes and treatments for each compartment. A**, Primary root; **B**, Lateral root; **C**, Cortex tissue; **D**, Stele. *rum1*, *rootless with undetectable meristem 1*; *rtcs*, *rootless concerning crown and seminal roots*; *lrt1*, *lateral rootless 1*; *rth*, *roothairless*. The explained variance by genotype, nutrient treatment condition and interaction were assessed by permutational analysis of variance (PERMANOVA, *P* <0.001).


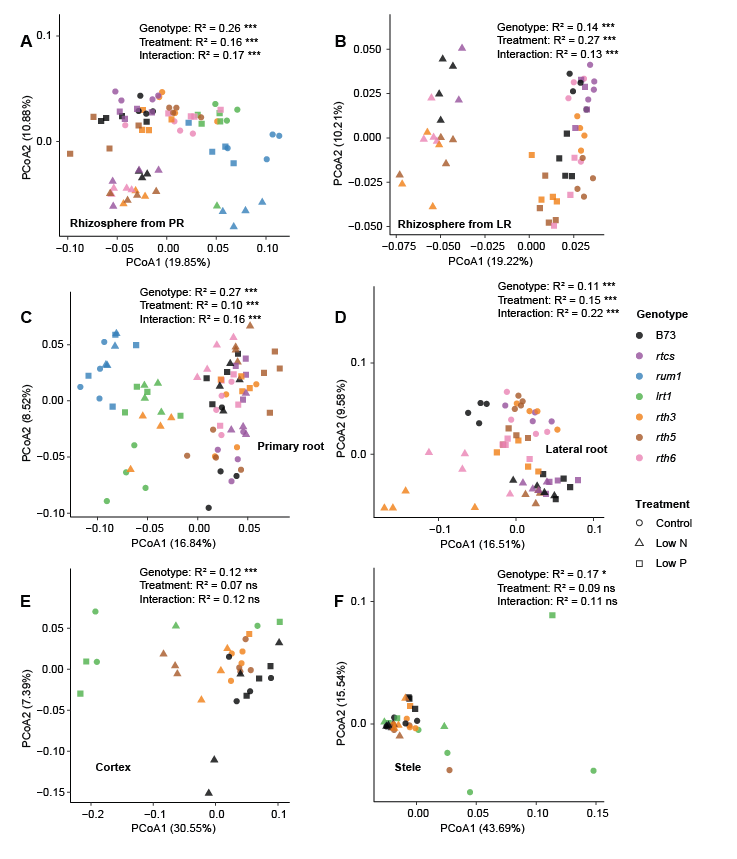


**Fig. S4. Principal coordinate analysis (PCoA) showing the dissimilarity of bacterial β-diversity for each compartment.** **A**, Rhizosphere from primary root; **B**, Rhizosphere from lateral root; **C**, Primary root; **D**, Lateral root; **E**, Cortex tissue; **F**, Stele. *rum1*, *rootless with undetectable meristem 1*; *rtcs*, *rootless concerning crown and seminal roots*; *lrt1*, *lateral rootless 1*; *rth*, *roothairless*. The explained variance by genotype, nutrient treatment condition and interaction were assessed by permutational analysis of variance (PERMANOVA, *P* <0.001).


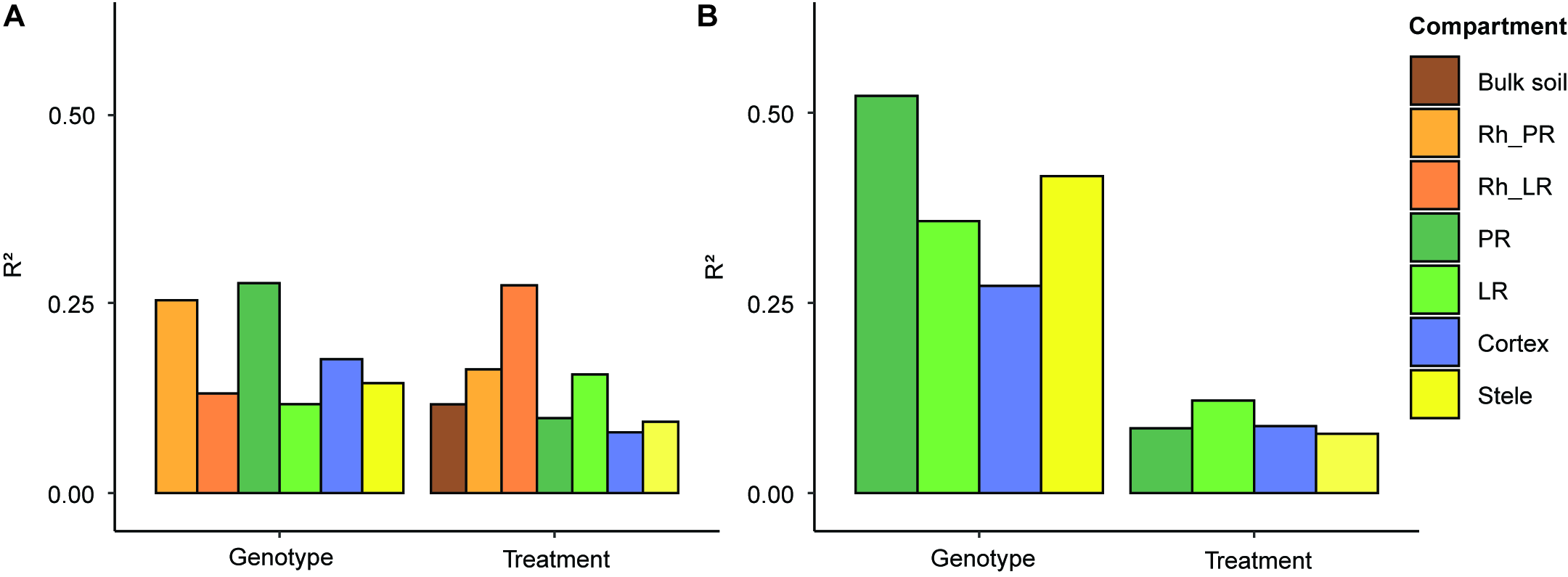


**Fig. S5. PERMANOVA results for PCoA of bacterial community composition and PCA of gene expression.** Rh_PR, Rhizosphere from primary root; Rh_LR, Rhizosphere from lateral root; PR, Primary root; LR, Lateral root.


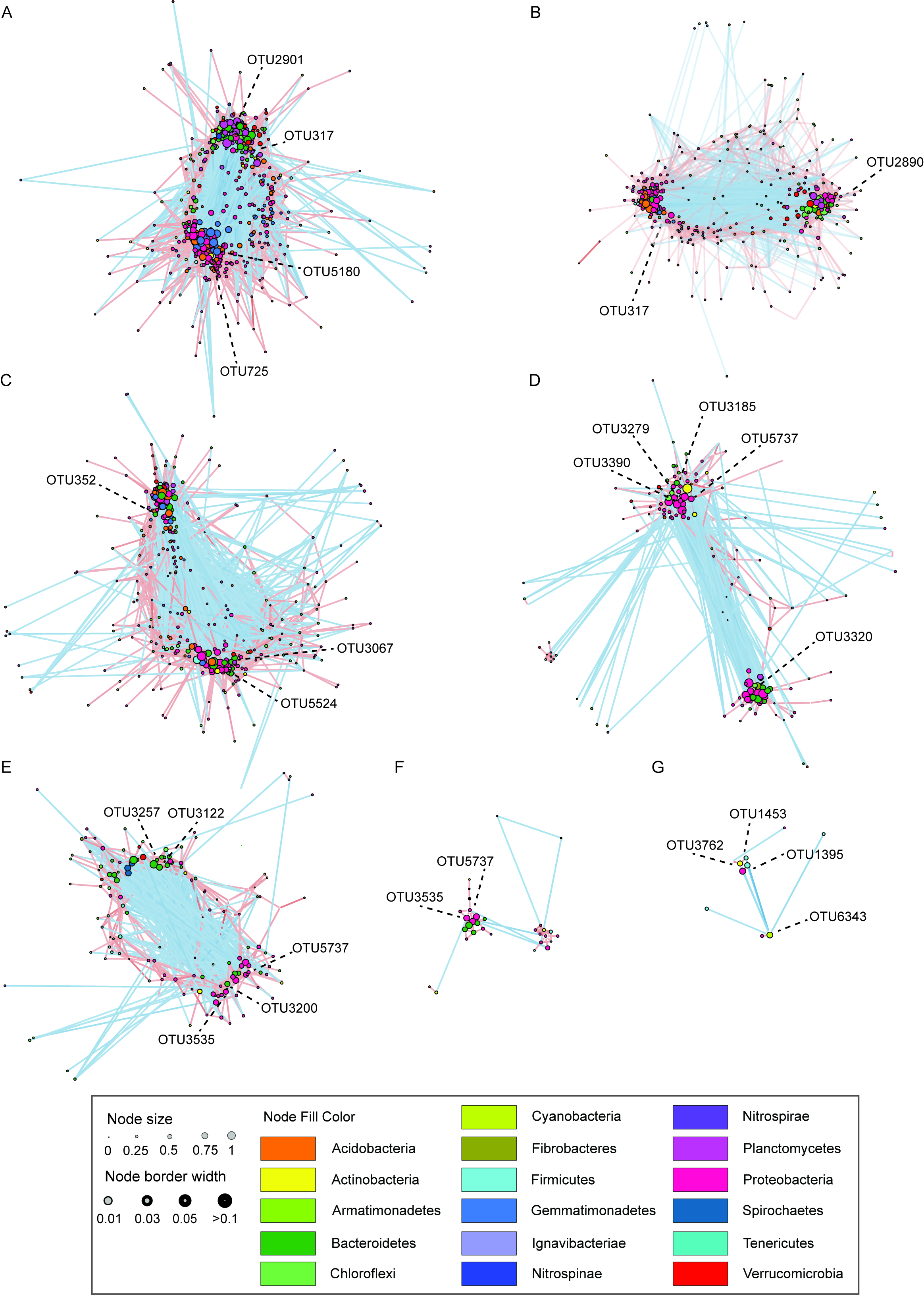


**Fig. S6 OTU-OTU co-occurrence network in soil (A), rhizosphere of primary root (B), rhizosphere of lateral root (C), primary root (D), lateral roots (E), cortex (F) and stele (G).** Nodes color represents phylum, node size is proportional to hub score and node border width is proportional to mean relative abundance. Key OTUs are labeled by OTU id. Red and blue solid lines indicate positive and negative correlations respectively.

**Fig. S7**. **Number of nodes and edges of OTU-OTU SparCC network within each compartment.** Rh_PR, Rhizosphere from primary root; Rh_LR, Rhizosphere from lateral root; PR, Primary root; LR, Lateral root.


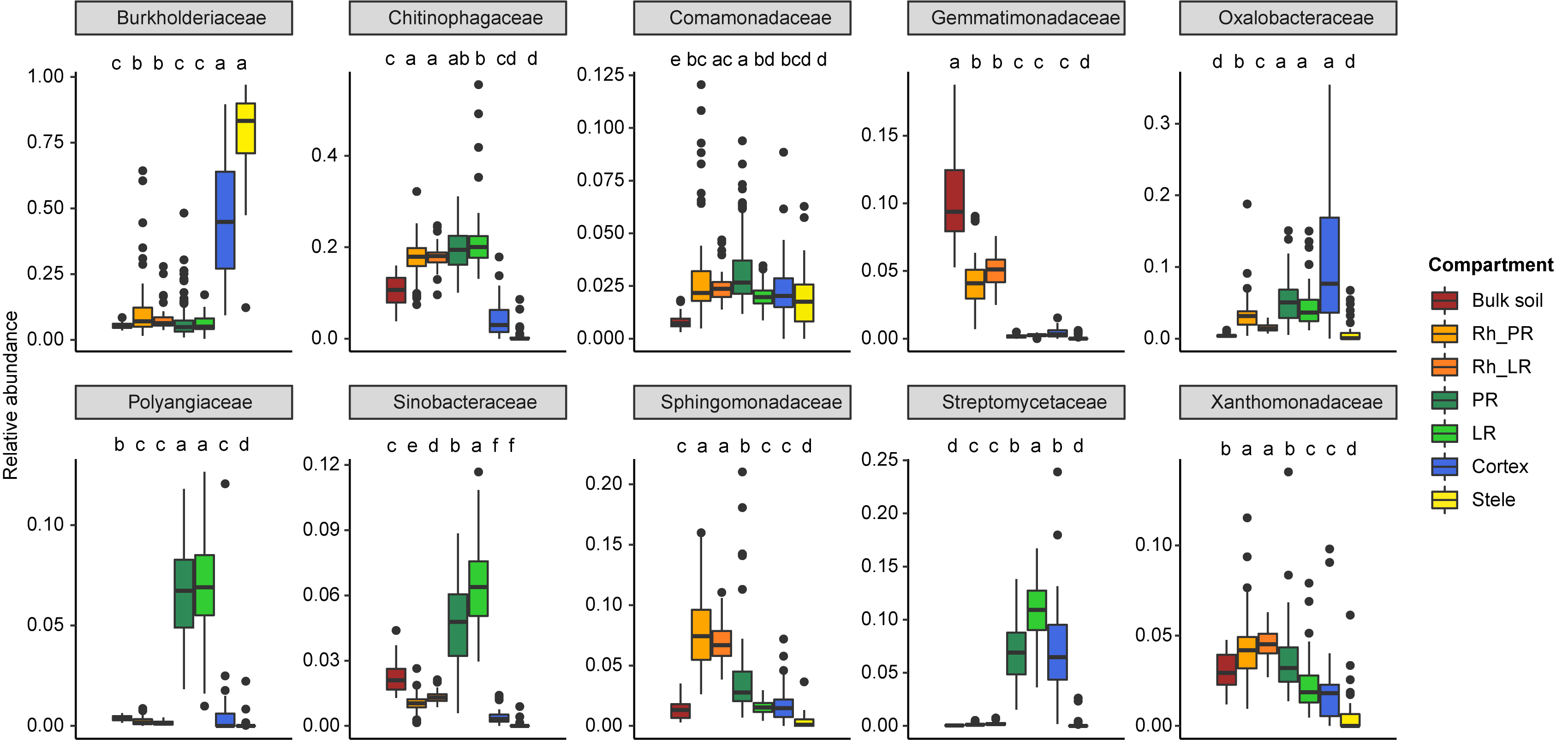


**Fig. S8.** **Relative abundance of top ten rich families across different compartments.** Rh_PR, Rhizosphere from primary root; Rh_LR, Rhizosphere from lateral root; PR, Primary root; LR, Lateral root. Significances were indicated among different compartments by different letters for each family (Benjamini-Hochberg adjusted *P* < 0.05, Kruskal-Wallis test, Dunn’s *post*-*hoc* test).


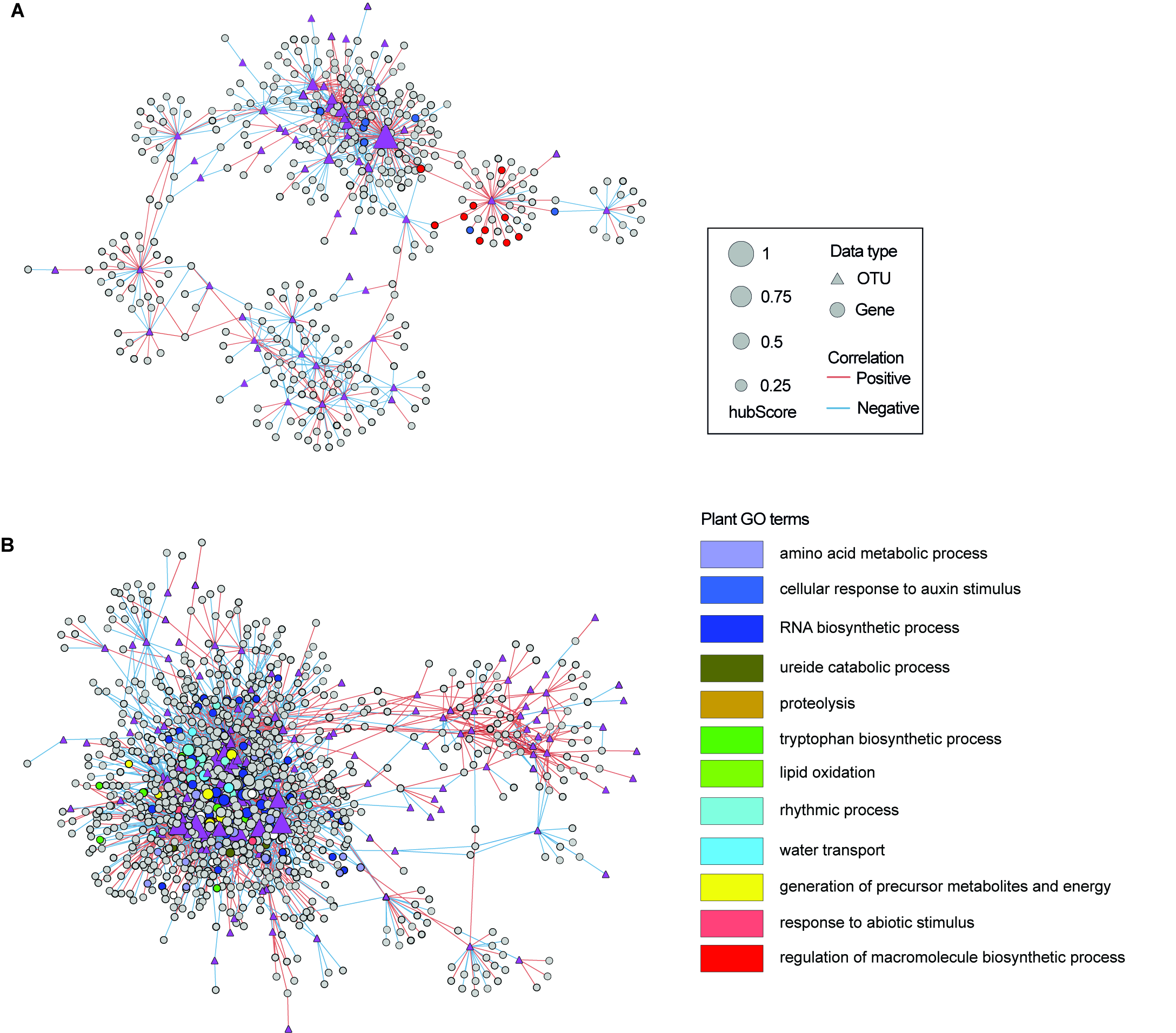


**Fig. S9. Network associations between plant genes and microbial OTUs in the rhizosphere from primary root (A) and lateral root (B).** The triangles and dots indicated the bacterial OTUs and gene features respectively. The size of the triangles indicates the hub score. Red and blue solid lines indicate positive and negative correlations respectively. Only the hub OTUs connected with genes with significant plant gene ontology (GO) terms are labelled accordingly.


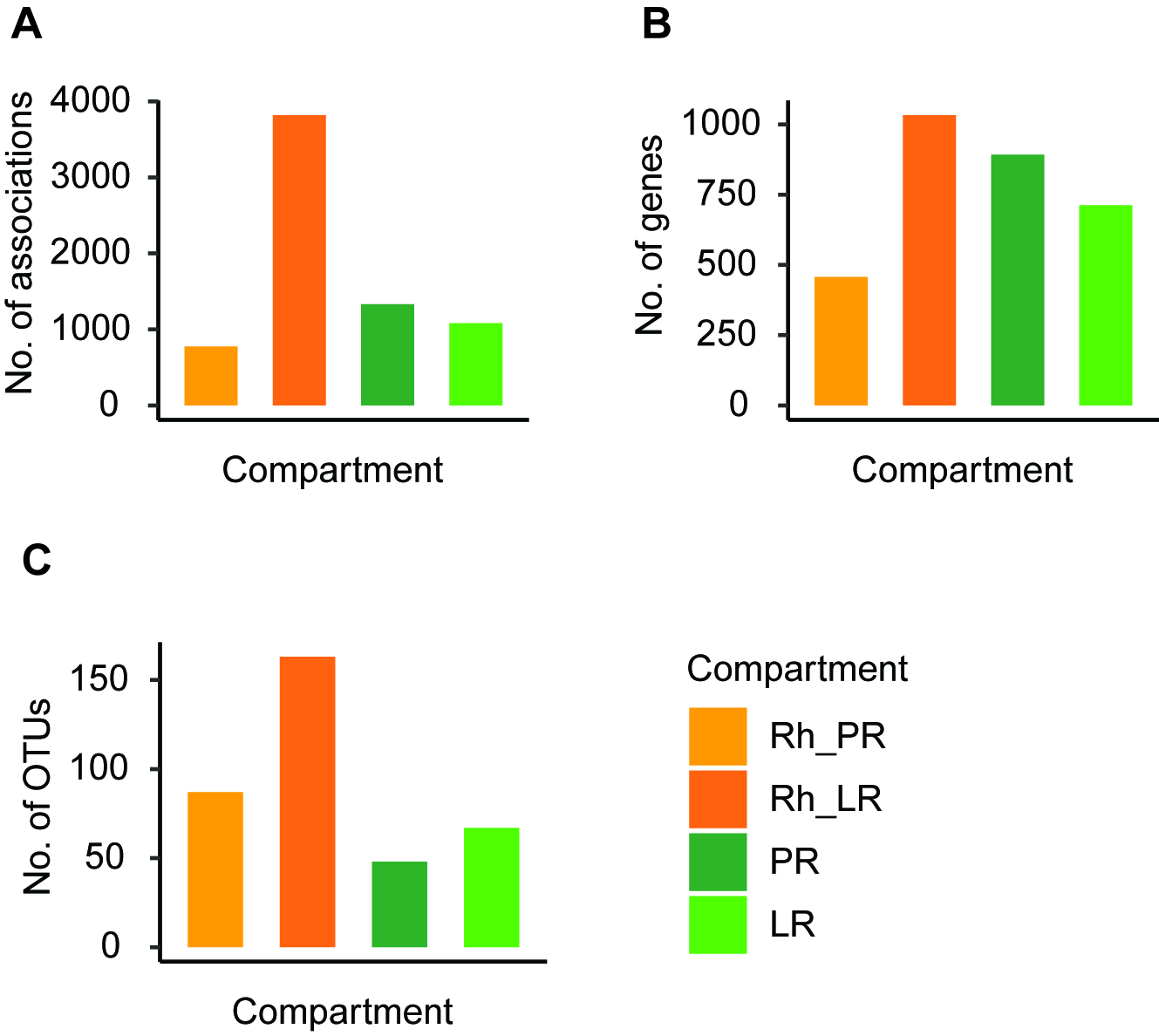


**Fig. S10. Number of edges, genes, and OTUs for each OTU-Gene network.** Rh_PR, Rhizosphere from primary root; Rh_LR, Rhizosphere from lateral root; PR, Primary root; LR, Lateral root.


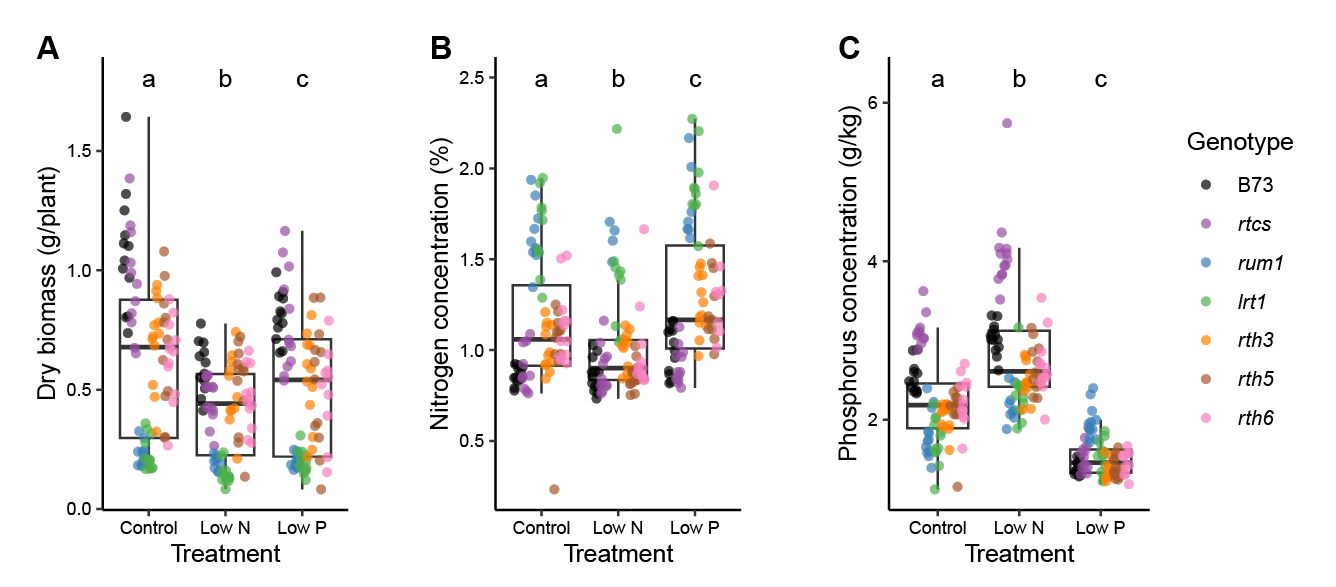


**Fig. S11. Maize phenotypic traits under different treatments.** **A**, Shoot dry biomass; **B**, Nitrogen concentration; **C**, Phosphorus concentration. N, nitrogen; P, phosphorus. Samples are colored in genotypes. Different letters indicate significant differences controlled by One-Way ANOVA at the *P* <0.05.


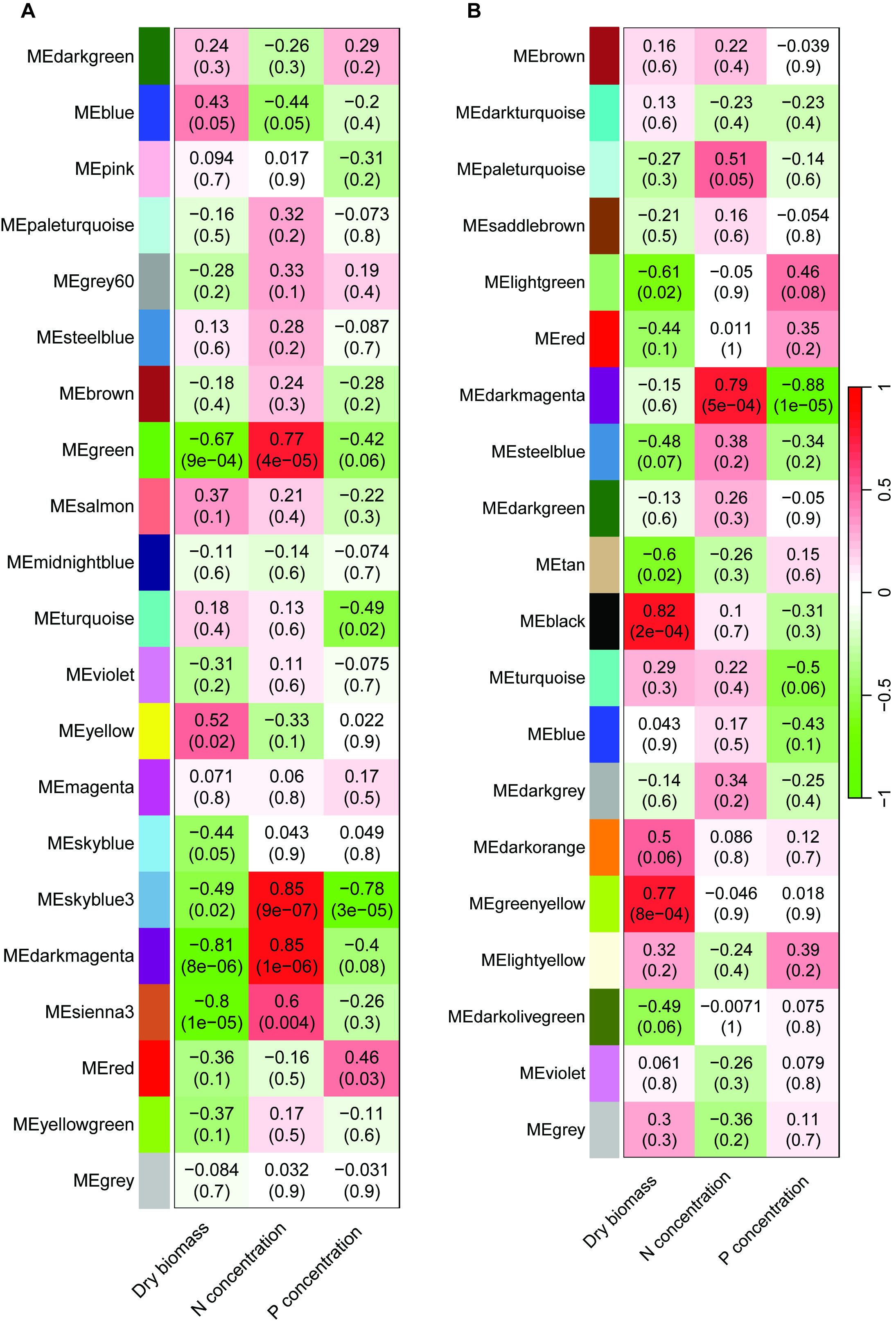


**Fig. S12.** **Gene WGCNA modules and their correlations with plant traits. A**, Primary root; **B**, Lateral root.


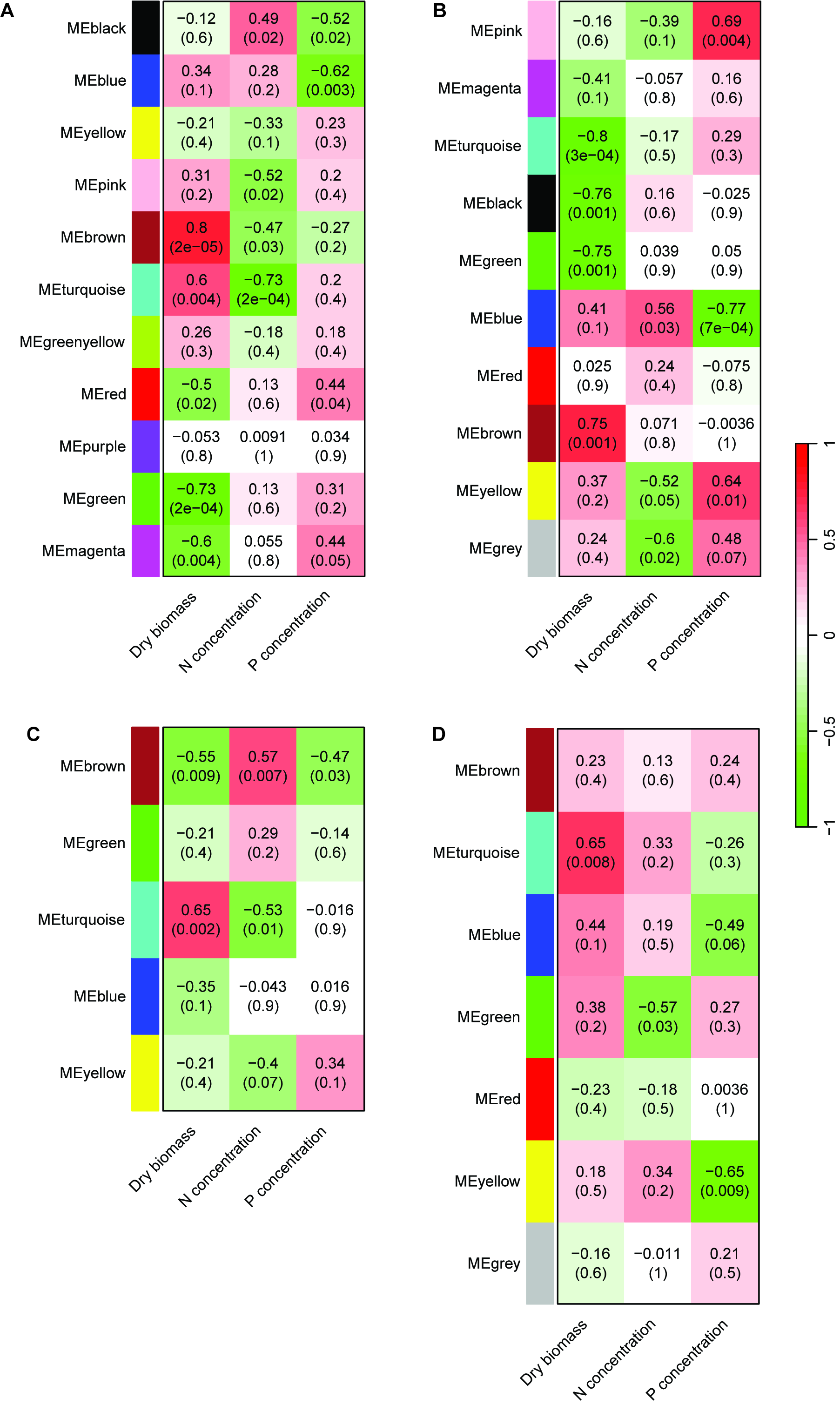


**Fig. S13. Bacterial WGCNA modules and their correlations with plant traits. A**, Rhizosphere from primary root; **B**, Rhizosphere from lateral root; **C**, Primary root; **D**, Lateral root.

**
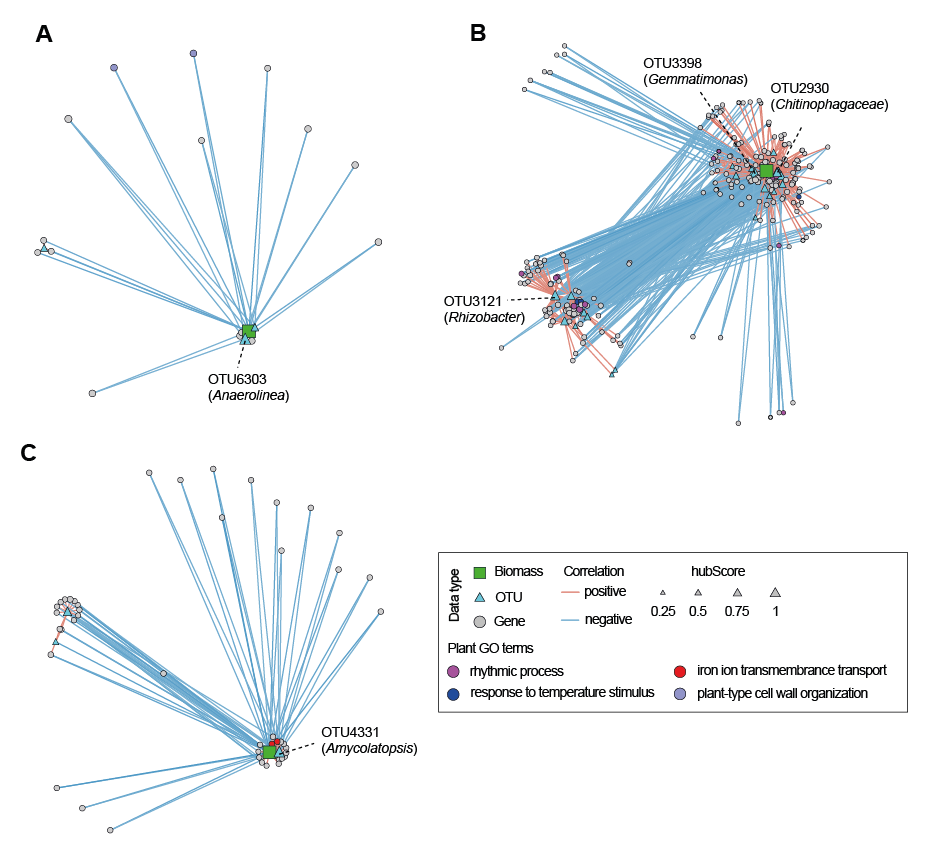
**

**Fig. S14. Bacterial hubs prioritize the causal association with plant rhythmic process and biomass accumulation.** Spearman correlation relationships between gene expression, OTU abundance and shoot biomass in the rhizosphere from primary root (**A**), rhizosphere from lateral root (**B**) and primary root (**C**). The triangles, dots and cubes indicated the microbiome, transcriptome and biomass features respectively. Red and blue solid lines indicate positive and negative correlations respectively. The actual length of the edge measured as the Euclidean distance between the source node and the target node. The size of the triangles indicates the hub score. Different color dots indicate specific plant gene ontology (GO) terms. The genus name of the hub OTUs and specific genes with GO annotation were labelled.

**Fig. S15. linear model between shoot dry biomass and OTU3535 relative abundance (%).** Linear model was fitted using lm() function.


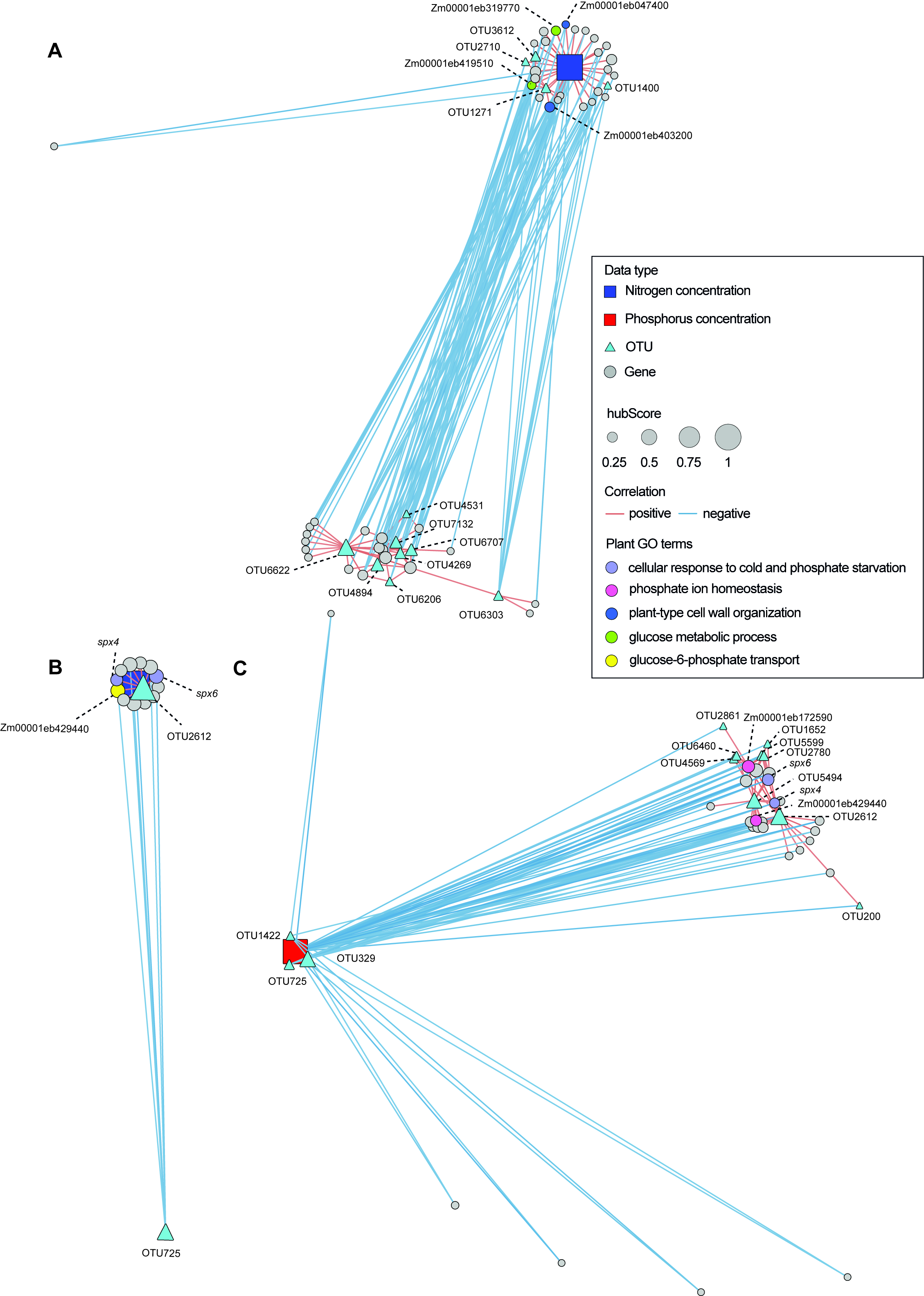


**Fig. S16. Trans-kingdom interaction network between bacterial OTUs and root genes in association with plant nutrients concentration. A**, Root gene expression, bacterial OTUs in the rhizosphere from primary root and nitrogen concentration; **B**, Root gene expression, bacterial OTUs in the rhizosphere from lateral root and nitrogen concentration; **C**, Root gene expression, bacterial OTUs in the rhizosphere from lateral root and phosphorus concentration.

**Fig. S17. PCA plot and differential expression genes between flowering stage and seedling stage.** PERMANOVA test was performed to calculate the variance explained by stage in gene expression (permutations = 1999). Differential expression genes were determined by set absolute value of log_2_Foldchange >2 and FDR adjusted *P* <0.01.
